# Homodimerization of HYL1 ensures the correct selection of cleavage sites in primary miRNA

**DOI:** 10.1093/nar/gku907

**Published:** 2014-10-07

**Authors:** Xi Yang, Wenqing Ren, Qiuxia Zhao, Peng Zhang, Feijie Wu, Yuke He

**Affiliations:** National Key Laboratory of Plant Molecular Genetics, Shanghai Institute of Plant Physiology and Ecology, Shanghai Institutes for Biological Sciences, Chinese Academy of Sciences, Shanghai 200032, China

## Abstract

MicroRNA (miRNA) plays an important role in the control of gene expression. HYPONASTIC LEAVES1 (HYL1) is a double-stranded RNA-binding protein that forms a complex with DICER-LIKE1 (DCL1) and SERRATE (SE) to process primary miRNA (pri-miRNA) into mature miRNA. Although HYL1 has been shown to partner with DCL1 to enhance miRNA accuracy, the mechanism by which HYL1 selects the DCL1-targeted cleavage sites in pri-miRNA has remained unknown. By mutagenesis of HYL1 and analysis of *in vivo* pri-miRNA processing, we investigated the role of HYL1 in pri-miRNA cleavage. HYL1 forms homodimers in which the residues Gly^147^ and Leu^165^ in the dsRBD2 domain are shown to be critical. Disruption of HYL1 homodimerization causes incorrect cleavage at sites in pri-miRNA without interrupting the interaction of HYL1 with DCL1 and accumulation of pri-miRNAs in HYL1/pri-miRNA complexes, leading to a reduction in the efficiency and accuracy of miRNAs that results in strong mutant phenotypes of the plants. HYL1 homodimers may function as a molecular anchor for DCL1 to cleave at a distance from the ssRNA–dsRNA junction in pri-miRNA. These results suggest that HYL1 ensures the correct selection of pri-miRNA cleavage sites through homodimerization and thus contributes to gene silencing and plant development.

## INTRODUCTION

MicroRNAs (miRNAs) are ∼21 nucleotide (nt) small RNAs produced from partially paired stem-loop regions of primary miRNA (pri-miRNA) transcripts (1). miRNAs regulate gene expression at the post-transcriptional level by inhibiting the expression of mRNAs bearing fully or partly homologous target sequences (1–3). The roles of many miRNAs in developmental regulation, disease resistance, and biotic and abiotic stress response have been demonstrated (4–6). To further understand the molecular mechanism of miRNA regulatory function, it is important to know how miRNA is processed from pri-miRNA.

In animals, pri-miRNAs are processed into miRNA precursors (pre-miRNAs) in nucleus by an RNaseIII-like enzyme named Drosha (7) that selectively cleaves RNA hairpins bearing a large terminal loop (8). After export to the cytoplasm by Exportin-5 (9,10), the Drosha homolog Dicer cleaves the pre-miRNAs into the miRNA:miRNA* duplex (11). In plants, DICER-LIKE1 (DCL1; the Arabidopsis homolog of Dicer) cleaves both pri-miRNA and pre-miRNA and generates miRNA in the nucleus (12,13), and then the miRNAs are exported to the cytoplasm and incorporated in the RNA-induced silencing complex (RISC) for negatively regulation to gene expression through mRNA degradation or translation inhibition (13–18).

DCL1, HYPONASTIC LEAVES1 (HYL1) and SERRATE (SE) are all required for the biogenesis of miRNAs (12,19–22) and function together in dicing bodies (D-bodies) (23). DCL1 catalyzes the processing of pri-miRNAs and pre-miRNAs, consequently producing mature miRNAs (12), whereas HYL1 and SE act as functional partners of DCL1 in the miRNA biogenesis machinery. *In vitro* miRNA processing assay and *in vivo* small RNA deep sequencing show that in the absence of HYL1, DCL1 alone generates more inaccurate miRNAs (19,24). There are some other new proteins that participate in the biogenesis of miRNAs. DAWDLE, a forkhead-associated domain-containing protein, facilitates DCL1 access to or recognition of pri-miRNAs (25). The cap-binding protein complex (CBC) can interact with the 5′ cap of pri-miRNA and affect the loading of pri-miRNAs to dicing complexes with SE (26). HUA ENHANCER 1 (HEN1) methylates miRNA/miRNA* duplexes, protecting them from degradation (27). MOS2 promotes pri-miRNA processing via facilitating the recruitment of pri-miRNAs by the dicing complexes (28). C-TERMINAL DOMAIN PHOSPHATASE-LIKE 1 (CPL1) is required for HYL1 dephosphorylation, which in turn is essential for accurate miRNA processing and strand selection (29).

HYL1 contains two double-stranded RNA-binding domains (dsRBDs) at its N-terminal end, a putative protein–protein interaction domain at its C-terminal end, and a nuclear localization signal (NLS) in the middle (30,31). The loss-of-function mutant *hyl1* exhibits pleiotropic phenotypes. The two N-terminal dsRBDs of HYL1 are sufficient to rescue the phenotypes by restoring the accumulation of miRNAs (31). HYL1 has been reported to regulate the phase transition, establishment of stamen, and the adaxial–abaxial identity of leaf in *Arabidopsis* by controlling the biogenesis of different miRNA families (32–34). Recently, the crystal structures of dsRBD1 and dsRBD2 of HYL1 have been reported (35). Based on their structures, dsRBD1 likely binds to RNAs and dsRBD2 may be responsible for protein interactions. Structural analyses also imply that HYL1 forms a dimer and that this dimerization is probably mediated by the dsRBD2 domain. However, the function of the dimer remains unknown.

In animals, several double-stranded RNA-binding proteins are involved in the two steps of miRNA processing. In the nucleus, DIGEORGE SYNDROME CRITICAL REGION GENE 8 (DGCR8) protein (also known as Pasha in *Drosophila melanogaster*) functions as the essential cofactor of Drosha (36–39). In the cytoplasm, TAR-RNA BINDING PROTEIN (TRBP) and LOQUACIOUS (LOQS) act as the partners of Dicer (31,40). TRBP contributes to RNA binding, length determination and the assembly of RISCs (41,42). Moreover, TRBP is required for optimal RNA silencing mediated by siRNAs and endogenous miRNAs, and it facilitates cleavage of pre-miRNA *in vitro* (43). LOQS is known to change the cleavage site targeted by Dicer to produce miRNAs with target specificities different from those generated by Dicer alone or Dicer bound to alternative protein partners (40).

Although several studies have demonstrated the contributions of HYL1 in the accuracy and efficiency of miRNA processing (19,44), the mechanism by which HYL1 recognizes primary RNA substrates and selects cleavage sites has yet to be elucidated. Here, we report that HYL1 regulates miRNA processing via homodimerization mediated by the dsRBD2 domain. Two residues, Gly^147^ and Leu^165^, play key roles in HYL1 dimerization. Disruption of HYL1 dimerization does not impair its binding to DCL1, SE, or the accumulation of pri-miRNA in dicing complexes, but it does interfere with miRNA biogenesis and plant development by altering the first cleavage sites on pri-miRNAs, which reduces the accuracy and efficiency of miRNA processing. HYL1 homodimers are responsible for the correct selection of cleavage sites in pri-miRNA because they determine the correct distance of cleavage sites from the ends of stem.

## MATERIALS AND METHODS

### Plant materials and growth conditions

*Arabidopsis thaliana* wild-type and *hyl1-2* (SALK_064863, Columbia ecotype) and *hyl1-3* (Columbia ecotype) mutants were used in this study. The *hyl1-3* mutants were obtained from Dr Detlef Weigel (29).

Seeds were surface-sterilized in 70% ethanol for 30 s and then in 0.1% HgCl_2_ for 10 min and washed four times in sterile distilled water. For *in vitro* tissue culture, the seeds were mixed in molten 0.1% water agar and plated on top of solid 1% sugar Murashige and Skoog medium. Plates were sealed with parafilm, incubated at 4°C in darkness for 2 days, and then moved to a growth chamber at 22.8°C for 16 h in light. For phenotypic observation, seeds were sown in pots with peat soil and grown in growth chambers in the same conditions as described above.

### Gene cloning and transgenes

A *HYL1* promoter region (1240 bp upstream of the translation start site) and a full-length coding sequence (1257 bp) were amplified from Columbia seedlings. Then both sequences were cloned into pCAMBIA1301 binary vectors to obtain the *pHYL1::HYL1* constructs. Site-directed mutagenesis was performed, and the primers used for polymerase chain reactions (PCRs) are listed in Supplementary Table S3.

The *Arabidopsis* plants were transformed using a ﬂower-dip method. For selection of transgenic plants, the seeds were sterilized and germinated on agar medium containing 50 mg/ml hygromycin. Seedlings conferring resistance to the hygromycin were transplanted in a greenhouse and grown at 22°C under an 8-h light regimen. The transgenic plants were shelved for at least three generations, and the seeds from each plant were harvested separately for subsequent observations.

### RNA analysis

For northern blotting, RNA samples were extracted using TRIzol reagent (Invitrogen, Carlsbad, CA, USA). For northern blotting of small RNA, 50 μg of total RNA was resolved by 15–19% polyacrylamide gel electrophoresis (PAGE) in 1× TBE at 80 V for 4–6 h and transferred to a Hybond membrane (Amersham Biosciences, GE Healthcare) in 0.5× TBE overnight at 28 mA. The UV cross-linked membrane was hybridized in ULTRAhyb® Ultrasensitive Hybridization buffer (Ambion, Austin, TX, USA) using probes of 3′ biotin-labeled DNA oligos (TaKaRa, Otsu, Japan) antisense to the mature miRNA or U6 transcripts (Supplementary Table S3). RNA gel blot analysis was performed according to the methods of Liu *et al.* (34).

For real-time PCR, total RNA was treated with DNase I (TaKaRa), followed by a phenol/chloroform extraction to remove contaminating DNA. Approximately 4 μg of purified RNA was used for first-strand complementary DNA (cDNA) synthesis using PrimeScript® Reverse Transcriptase (TaKaRa) with oligo(dT) primers. Real-time PCR was performed using the specific primer pairs (Supplementary Table S3) in the MyiQ2 Two-color Real-time PCR Detection System (Bio-Rad, Hercules, CA, USA). The comparative threshold cycle method was used to determine relative transcript levels. Quantitative PCR for each gene was done with at least three biological replicates.

### Analysis of transcriptional activity

Primary mRNAs of some *MIRNA* genes that contain introns were analyzed to evaluate transcriptional activity according to the methods of Liu *et al.* (45). The intron-specific primers and oligo(dT) were used for reverse transcription, and then the intron-specific primers for detection of primary mRNA levels of *MIRNA* genes (Supplementary Table S3).

### Protein analysis

Anti-Flag (Sigma-Aldrich, St Louis, MO, USA; F3165, 1:5000 dilution), anti-GST (Sigma-Aldrich; 1:5000 dilution), anti-MBP (NEB, 1:3000 dilution) and anti-HYL1 (Agrisera, 1:1000 dilution) antibodies were used for western blotting. Secondary antibodies were goat-developed anti-rabbit IgG (GE Healthcare; NA931V, 1:20 000 dilution) and goat-developed anti-mouse IgG (GE Healthcare; NA934V, 1:50 000 dilution).

### Coimmunoprecipitation

For coimmunoprecipitation assays of HYL1, plants of 10-day-old seedlings were ground into a ﬁne powder using liquid nitrogen and then resolved in a buffer containing 20 mM Tris-HCl, pH 7.4, 200 mM KCl, 40 mM MgCl_2_, 1 mM DTT, 20% glycerol and 0.05% Nonidet P-40. After pre-clearing with protein G-agarose beads for 1 h at 4°C, the extracts were incubated with HYL1 antibodies to protein G-agarose beads for 4 h at 4°C. After five washes (20 mM Tris-HCl, pH 7.9, 0.5 M KCl, 10% glycerol, 1 mM EDTA, 5 mM DTT, 0.5% Nonidet P-40 and 0.2 mM phenylmethylsulfonyl ﬂuoride), the proteins in the immunoprecipitates were subjected to northern and western blot analyses.

### Expression and purification of HYL1 proteins from *Escherichia coli*

For protein expression and purification, full-length and truncated *HYL1* was inserted into the expression vectors pGEX4T-1 and pMAL-C2x, respectively, and the recombinant plasmids were transformed into *E. coli* DH5α. Bacteria were cultured at 37°C overnight in a 20-ml volume, then diluted to 200 ml with 0.8 mM isopropylthiogalactoside and grown at 37°C for 4 h. Nearly 2 L of cultured bacteria was collected and lysed to generate ultrasonic precipitates, and the precipitates were then resolved in a buffer containing 1% Triton X-100, 20 mM Tris, 20 mM EDTA and 1 mM DTT. The GST-fused proteins were purified with glutathione Sepharose resin (GE Healthcare), whereas the MBP-tagged proteins were purified with Amylose Resin (NEB).

### Expression and purification of recombinant DCL1 and SE proteins from human embryonic kidney 293 cells (HEK293)

The cDNAs of DCL1 and SE with 3×Flag were cloned into pN3 vector (from pEGFP-N3 Clontech, but without an enhanced green fluorescent protein tag). The human embryonic kidney 293 (HEK293) cells were maintained in Dulbecco's modified Eagle's medium (Invitrogen) supplemented with 10% fetal bovine serum (FBS) (Gibco) and transfected with the DCL1 and SE vectors by Lipofectamine 2000 (Invitrogen) according to the manufacturer's protocol. The cells were harvested 48 h after transfection and lysed in lysis buffer (50 mM Tris-HCl, pH 7.4, 150 mM NaCl, 1 mM EDTA, 1 mM DTT, 0.5% Nonidet P-40, 0.2 μg/ml RNaseA, protease inhibitor cocktail) for 15 min. The lysate was then incubated with Flag resin in lysis buffer overnight at 4°C. After five washes with washing buffer (50 mM Tris HCl, 150 mM NaCl, pH 7.4, 0.005% NP-40), the Flag-fusion proteins were then eluted with 3×Flag peptide (150 ng/μl) in Tris-buffered saline (TBS) (50 mM Tris-HCl, pH 7.4, 150 mM NaCl).

### *In vitro* pull-down

For MBP pull-down, MBP-tagged proteins were bound to amylose resin (NEB) in binding buffer containing 25 mM Tris, pH 7.4, 1 mM EDTA, 0.01% NP-40 and 2 M NaCl, and incubated with GST-tagged proteins overnight at 4°C. Then the resin was washed 10 times in the binding buffer and eluted by boiling in sodium dodecyl sulfate (SDS)-PAGE loading dye. Aliquots of eluents (20 μl) were resolved on SDS-PAGE gels for immunoblotting with GST antibody.

### Yeast two-hybrid assays

Yeast transformation, mating and cDNA library preparation were performed according to the Matchmaker™ Gold Yeast Two-Hybrid System User Manual (Clontech). For the initial screen, a pGBKT7+ bait was transformed into Y2H Gold (Clontech) and then mated with the pistil library. Mating mixtures were screened on yeast media lacking -Leu-Trp-Ade+3AT. Inserts were amplified from yeast cultures using colony PCR followed by direct sequencing.

The fragments detected were fused in frame with the GAL4 DNA binding domain (pGBKT7) or activation domain (pGADT7). All the recombinant plasmid pairs were cotransformed into the yeast strain AH109. Yeast transformation was performed following the manufacturer's instructions (Clontech Yeast Protocols Handbook). The cotransformed yeast clones were first grown on SD/-Leu/-Trp medium and subsequently plated on SD/-Ade/-His/-Leu/-Trp medium.

### Bimolecular fluorescence complementation

Paired cYFP-tagged and nYFP-tagged constructs were cotransformed into *Arabidopsis* protoplasts. After incubation at 22.5°C in darkness for 12 h, yellow fluorescent protein (YFP) fluorescence signals and chlorophyll autofluorescence signals were excited at 658 nm and detected by confocal microscopy.

### Pri-miRNA processing *in vitro*

RNA substrates were transcribed under the T7 promoter *in vitro* using PCR-generated templates. The *in vitro* transcription of RNAs was carried out 3 h or overnight at 37°C in one 20-μl reaction containing: 1 μl of DNA template (100 ng), 4 μl of 5× transcription buffer (400 mM HEPES, pH 7.5, 10 mM spermidine, 200 mM DTT, 125 mM MgCl_2_ and 20 mM of each NTP), 1 μl of RNase inhibitor (Ambion), 2 μl of T7 RNA polymerase and 12 μl of water. DNase-treated RNA was fractionated on a 6% polyacrylamide and 8 M urea gel (denaturing gel), eluted overnight from gel slices in RNA elution buffer (0.3 M NaAc, pH 5.5 and 2% SDS) using Thermomixer R (Eppendorf) at 4°C and 1200 rpm, precipitated with ethanol, and stored in diethylpolycarbonate water.

Briefly, each 10-μl RNA cleavage assay mixture contained 20 mM Tris HCl (pH 7.0), 50 mM NaCl, 4 mM MgCl_2_, 5 mM ATP, 1 mM GTP, 2 units of RNase inhibitor (TaKaRa), RNA substrate, and recombinant DCL1, HYL1 and SE proteins. For common reactions, the estimated concentration of proteins and substrate are: DCL1:50 mM; SE:150 mM; HYL1:100 mM; pri-miRNA:200 mM.

After incubation at 37°C for 30 min, the products were extracted with phenol/chloroform and precipitated. The processing products were fractionated by PAGE in a 12–19% acrylamide-8 M urea gel and detected by northern blotting.

### Cloning of RNA cleavage products

For *in vitro* processing, the bands corresponding to oligonucleotides in the vicinity of 21 nt were eluted from gel slices and coprecipitated with glycogen. A 5′ adaptor and a 3′ adaptor were ligated to the RNA cleavage products sequentially. Reverse transcription-PCR (RT-PCR) was carried out with the adaptor-ligated RNA cleavage products. PCR fragments were cloned into the pGEM-T-easy vector (Promega) and sequenced with M13F primer. Sequences were analyzed with Vector NTI software (Invitrogen).

For 5′ RACE PCR, RNA samples were isolated from the seedlings of Col, *hyl1-2* and *hyl1-3*. Hundred picomoles of 5′ adaptor (rGrUrUrCrArGrArGrUrUrCrUrArCrArGrUrCrCrGrArCrGrArUrC) were directly ligated with 5 μg of total RNA. After ligation, first-strand cDNAs were synthesized using Superscript III Reverse Transcriptase (Invitrogen) and an RNA-specific primer (5′ RACE outer reverse). The cDNA was treated with RNase H to remove the RNA strand and amplified in two rounds using two sets of primers (5′ RACE outer forward and 5′ RACE outer reverse, and 5′ RACE inner forward and 5′ RACE inner reverse). The distinct PCR products were cloned into the pGEM-T-easy vector (Promega) and sequenced using either M13 forward or M13 reverse primers.

## RESULTS

### HYL1 forms a homodimer by dsRBD2 domain

HYL1 acts as an important partner of DCL1 within the D-body. To gain insight into the components of the D-body necessary for accurate miRNA processing, we carried out a yeast two-hybrid screening of HYL1 to investigate the unidentified factors involved in the accuracy of miRNA processing. Several proteins including SE and some functionally unknown proteins were found to interact with HYL1 (Supplementary Table S1). Interestingly, we identified protein–protein interaction of HYL1 with itself, indicating that HYLl forms a homodimer.

**Figure 1.**
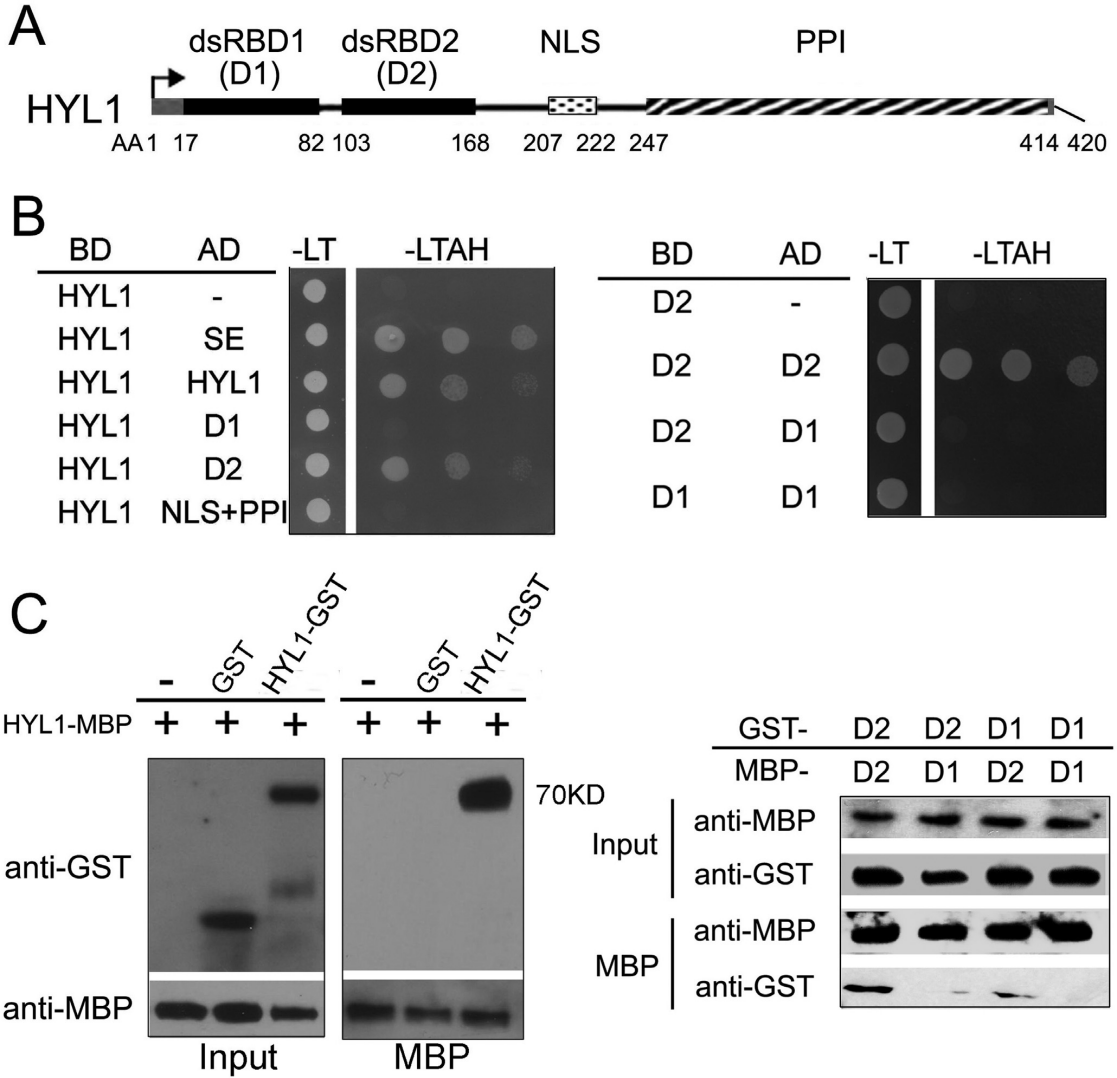
Homodimerization of HYL1 through dsRBD2. (**A**) Diagram of functional domains in HYL1 protein. dsRBD1, double-stranded RNA-binding domain 1; dsRBD2, double-stranded RNA-binding domain 2; NLS, nuclear localization signal; PPI, protein–protein interaction domain. (**B**) Homodimerization of HYL1 and HYL1 mutants in yeast two-hybrid assay. D1: dsRBD1; D2: dsRBD2; AD, GAL4 activation domain fusions; DB, GAL4 DNA binding domain fusions; -LT, medium without leucine and tryptophan; -LTAH, without leucine, tryptophan, adenine and histidine. Growth in the -LTAH medium indicates protein–protein interaction. (**C**) Pull-down assay results for HYL1 homodimers. D1, dsRBD1; D2, dsRBD2.

The two dsRBDs, putative protein–protein interaction domain and NLS of HYL1 are indicated in Figure 1A. To determine whether HYL1 forms a homodimer *in vitro*, we used full-length and truncated HYL1 for a yeast two-hybrid assay. We observed a direct interaction between two HYL1 molecules (Figure 1B). The dsRBD2 fragment interacted with full-length HYL1, whereas the dsRBD1 fragment did not. Moreover, one dsRBD2 fragment interacted with another dsRBD2 but not with dsRBD1. These findings indicate that HYL1 forms a homodimer through the dsRBD2 domains.

To further examine whether HYL1 homodimerizes through dsRBD2, we performed pull-down assays. As expected, MBP-HYL1 pulled down GST-HYL1, and MBP-dsRBD2 pulled down GST-dsRBD2, but MBP-dsRBD1 failed to pull down GST-dsRBD1 (Figure 1C; Supplementary Figure S1). In addition, weak interaction signals were seen between dsRBD1 and dsRBD2. These results confirm that HYL1 dimerizes through the dsRBD2 domains.

To determine which domain is necessary for the interactions of HYL1 with SE and DCL1, we performed yeast two-hybrid assay of truncated HYL1 with SE and DCL1. The dsRBD2 domain rather than dsRBD1 domain interacted with SE and DCL1 (Supplementary Figure S2). Thus, we conclude that the dsRBD2 domain is the core of the HYL1 homodimerization and protein interactions.

### Gly^147^ and Leu^165^ are required for HYL1 dimerization

To identify the residues required for homodimerization of HYL1, we accomplished an extensive mutagenesis analysis. Based on the structure of the HYL1 dsRBD2, we selected seven amino acids within the dsRBD2 domain to generate different HYL1 mutations (Supplementary Table S2) because they are located in the exposed regions that could be involved in HYL1 dimerization. Eight HYL1 mutations were generated and each HYL1 mutation was expected to potentially impair HYL1 dimerization. In addition to these mutations, three other mutations that may impair the interaction with SE and DCL1 were generated.

**Figure 2.**
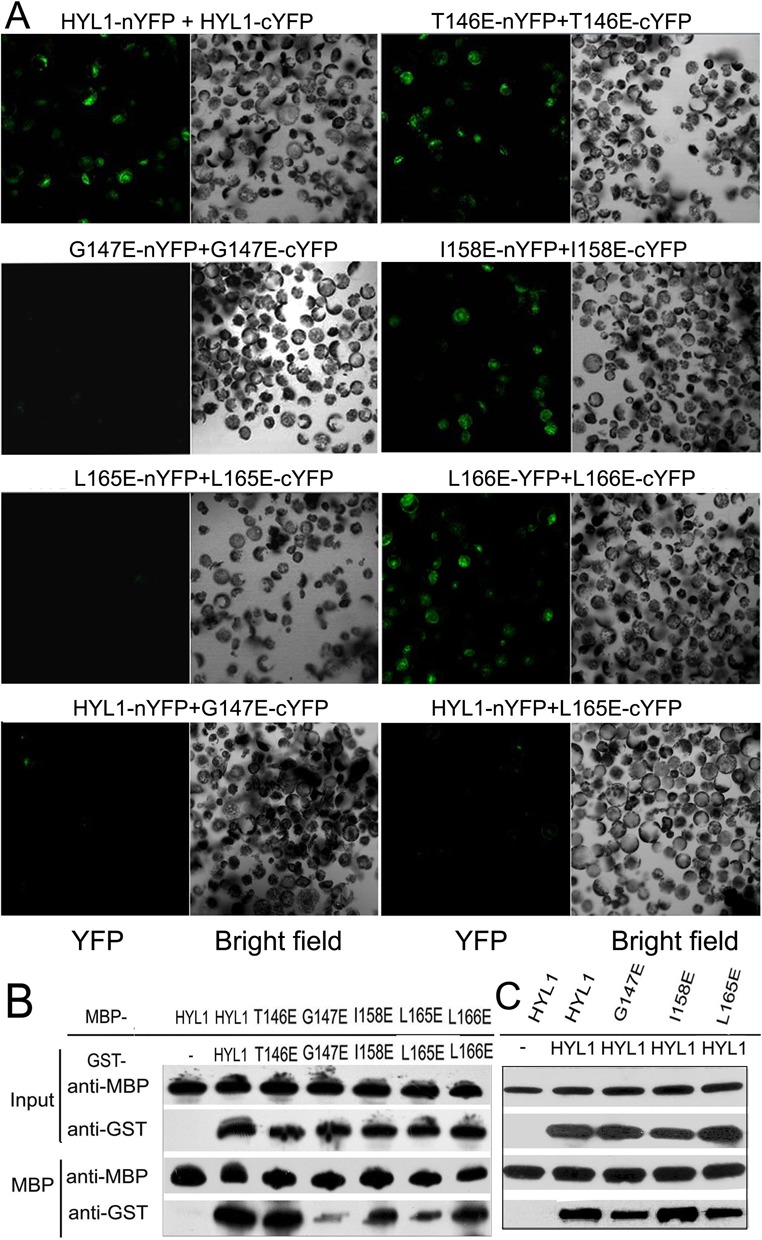
Crucial role of Gly^147^ and Leu^165^ in HYL1 dimerization. (**A**) BiFC analysis showing homodimerization of HYL1 and HYL1 mutants. (**B**) Pull-down assay results showing protein–protein interaction of HYL1 mutants with themselves. T146E, HYL1^T146E^; G147E, HYL1^G147E^; I158E, HYL1^I158E^; L165E, HYL1^L165E^; L166E, HYL1^L166E^. (**C**) Pull-down assay results showing protein–protein interaction of HYL1 mutants with wild-type HYL1.

Bimolecular fluorescence complementation (BiFC) was used to evaluate the effects of the point mutations on HYL1 dimerization. The same HYL1 mutants were fused with YFP at either the N- or C-terminus and introduced into the *Arabidopsis* protoplast for transient expression. The HYL1 mutants were visualized via confocal microscopy. Fluorescently labeled HYL1 mutants during the formation of BiFC complexes showed that the fluorescence intensity for HYL1^G147E^ (G147E) or HYL1^L165E^ (L165E) was significantly diminished, revealing that the homodimerization of these mutants are weakened (Figure 2A). By contrast, HYL1^C122R^ (C122R), HYL1^R130E^ (R130E), HYL1^T146R^ (T146R), HYL1^T146E^ (T146E), HYL1^I158E^ (I158E) and HYL1^L166E^ (L166E) showed almost the same fluorescence intensity as the wild-type HYL1 did, meaning that they also formed homodimers (Supplementary Figure S3A). Weaker fluorescence intensity was also found when G147E and L165E were cotransformed with wild-type HYL1. To exclude the possibility of transient transfer efficiency, western blotting was carried out to detect the mutated HYL1 fused with YFP. All five HYL1 mutants examined showed almost the same intensity of YFP (Supplementary Figure S4) in the protoplasts, demonstrating that the difference in transfer efficiency in the BiFC analysis was not significant. Therefore, we conclude that Gly^147^ and Leu^165^ are required for HYL1 dimerization. Using this method, we also examined the effects of the other three mutations (R151E, K154E and R162E) on their interaction with SE and DCL1, but found no difference from wild-type HYL1 (data not shown).

To confirm these findings, we used *in vitro* pull-down experiments to examine the associations of the HYL1 mutants. MBP-HYL1 pulled down GST-HYL1 as expected (Figure 2B). However, MBP-G147E and MBP-L165E pulled down much less GST-G147E and GST-L165E, respectively, and MBP-G147E and MBP-L165E pulled down less GST-HYL1 (Figure 2C). These results provide evidence that the Gly^147^ and Leu^165^ mutations impaired HYL1 dimerization.

### HYL1 dimerization is not necessary for direct interaction with DCL1 and SE

**Figure 3.**
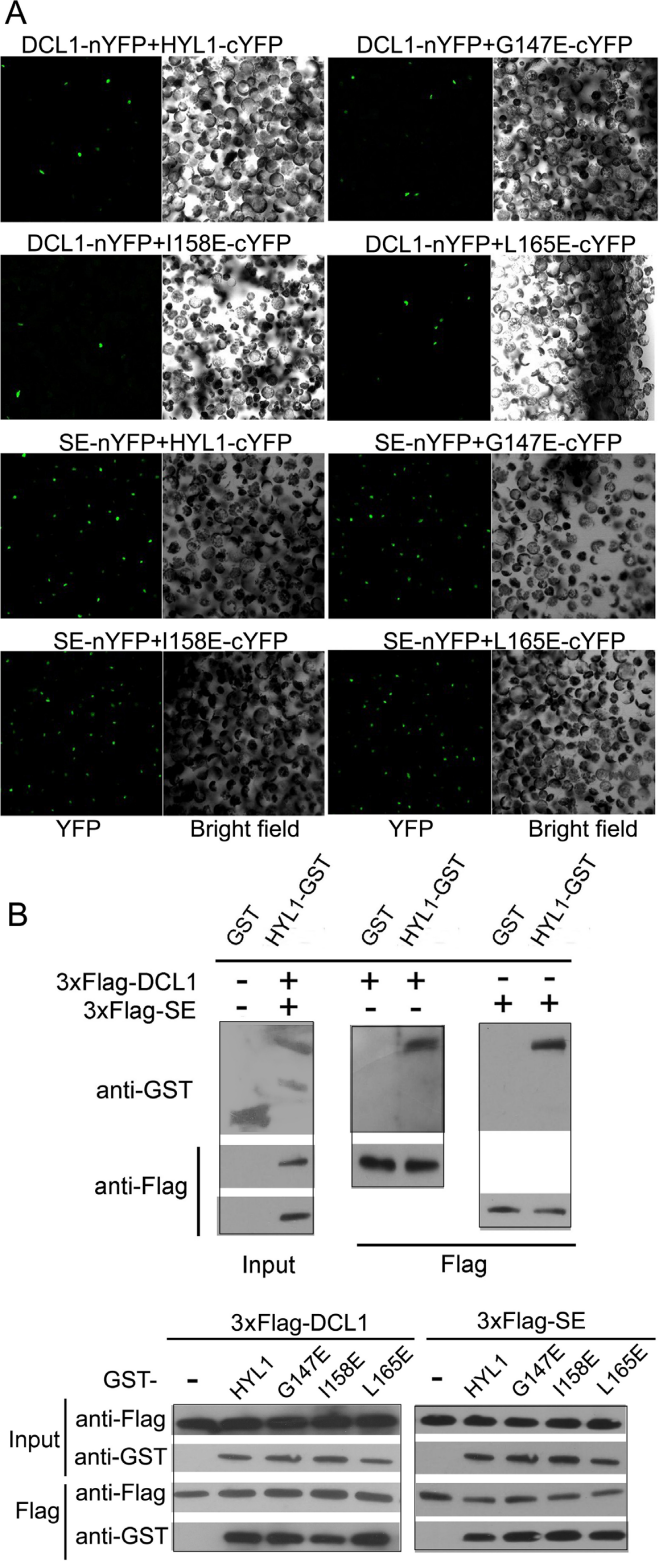
HYL1 dimerization is not necessary for direct interaction of HYL1 with DCL1 and SE. (**A**) BiFC analysis showing protein–protein interaction of HYL1 mutants with DCL1 and SE in protoplasts. (**B**) Pull-down assay results showing protein–protein interaction of HYL1 mutants with DCL1 and SE tagged with 3×Flag.

HYL1 is known to interact with DCL1 and SE, but whether non-dimerized HYL1 interacts with DCL1 and SE remains unknown. Therefore, we used BiFC analysis to determine the effects of the point mutations on their interactions with DCL1 and SE. In *Arabidopsis* protoplasts, all transiently expressed HYL1 mutants, including G147E and L165E, were found to interact with DCL1 and SE, implying that a deficiency in HYL1 dimerization did not affect protein–protein interactions of HYL1 with DCL1 and SE (Figure 3A; Supplementary Figure S3B).

To further test whether disruption of HYL1 homodimerization prevents the direct interaction of HYL1 with SE and DCL1 *in vitro*, we used pull-down assays to observe the interaction of G147E and L165E with DCL1 and SE. HYL1 was purified from recombinant proteins expressed in *Escherichia coli* as described previously (31). Recombinant DCL1 and SE were poorly expressed in *E. coli* but could be expressed in mammalian cells. Therefore, we obtained the recombinant DCL1 and SE proteins using HEK293 cells. DCL1 and SE tagged with 3×Flag were purified by Flag resin and then confirmed by Coomassie Blue staining (Supplementary Figure S5A). Western analysis showed that the strength of interactions between G147E and DCL1 and between L165E and DCL1 did not differ from that between HYL1 and DCL1 (Figure 3B). Similarly, the strength of interactions between G147E and SE and between L165E and SE was equal to that between HYL1 and SE. These results suggest that HYL1 homodimerization does not interfere with the direct interaction of HYL1 with DCL1 and SE.

### HYL1 homodimer is essential for normal plant development

**Figure 4.**
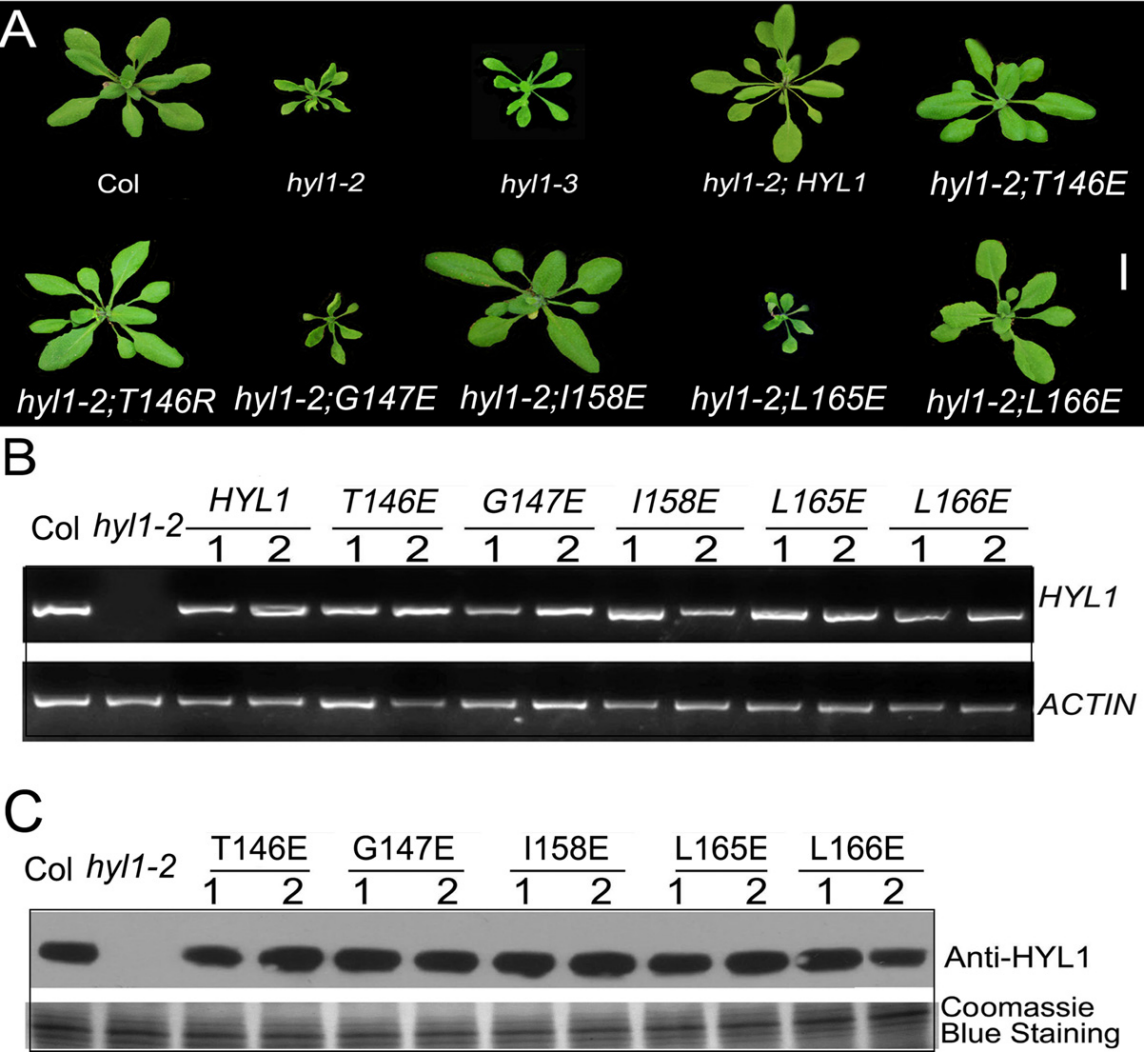
Functional analysis of HYL1 mutants in plant development. (**A**) Transgenic plants expressing HYL1 mutants at seedling stages. (**B**) The expression of *HYL1* gene in transgenic plants. (**C**) The protein levels of HYL1 mutants in transgenic plants.

To examine the biological functions of HYL1 homodimers, we constructed eight mutations of *HYL1* under the control of the native promoter and introduced them into the null *hyl1-2* mutants. *G147E* and *L165E* did not rescue the phenotypes of the *hyl1-2* mutants (Figure 4A), but *T146E*, *I158E* and the other mutations completely rescued the *hyl1-2* mutant phenotype in terms of leaf incurvature. These results indicated that *T146E* and *I158E* retained normal function, whereas *G147E* and *L165E* were deficient in some aspects of plant development. RT-PCR and western blotting showed that all HYL1 mutants in *hyl1-2* plants were expressed equally at both the transcriptional and translational levels (Figure 4B and C).

To define the disregulation of *G147E* and *L165E* to plant development, we searched for the mutants of *HYL1* gene that are related to these point mutations. The *hyl1-3* mutant firstly identified by Manavella *et al.* (29) was finally selected because the *hyl1-3* allele has a G-A mutation at position 440 of *HYL1* coding sequence, which causes a substitution at amino acid position 147. Actually, the *hyl1-3* allele shares a mutation at the same residue as in the transgenic line with *G147E*. In phenotype, the point-mutated *hyl1-3* plants looked similar to the null *hyl1-2* plants, as they showed the leaf incurvature, with slightly larger plants than the *hyl1-2* (Figure 4A). This phenotype suggests that the endogenous *G147E* in the *hyl1-3* mutant performs the same function as exogenous *G147E* in *G147E* plants.

### Impairment of HYL1 dimerization disrupts HYL1 function in miRNA biogenesis and miRNA-guided gene silencing

**Figure 5.**
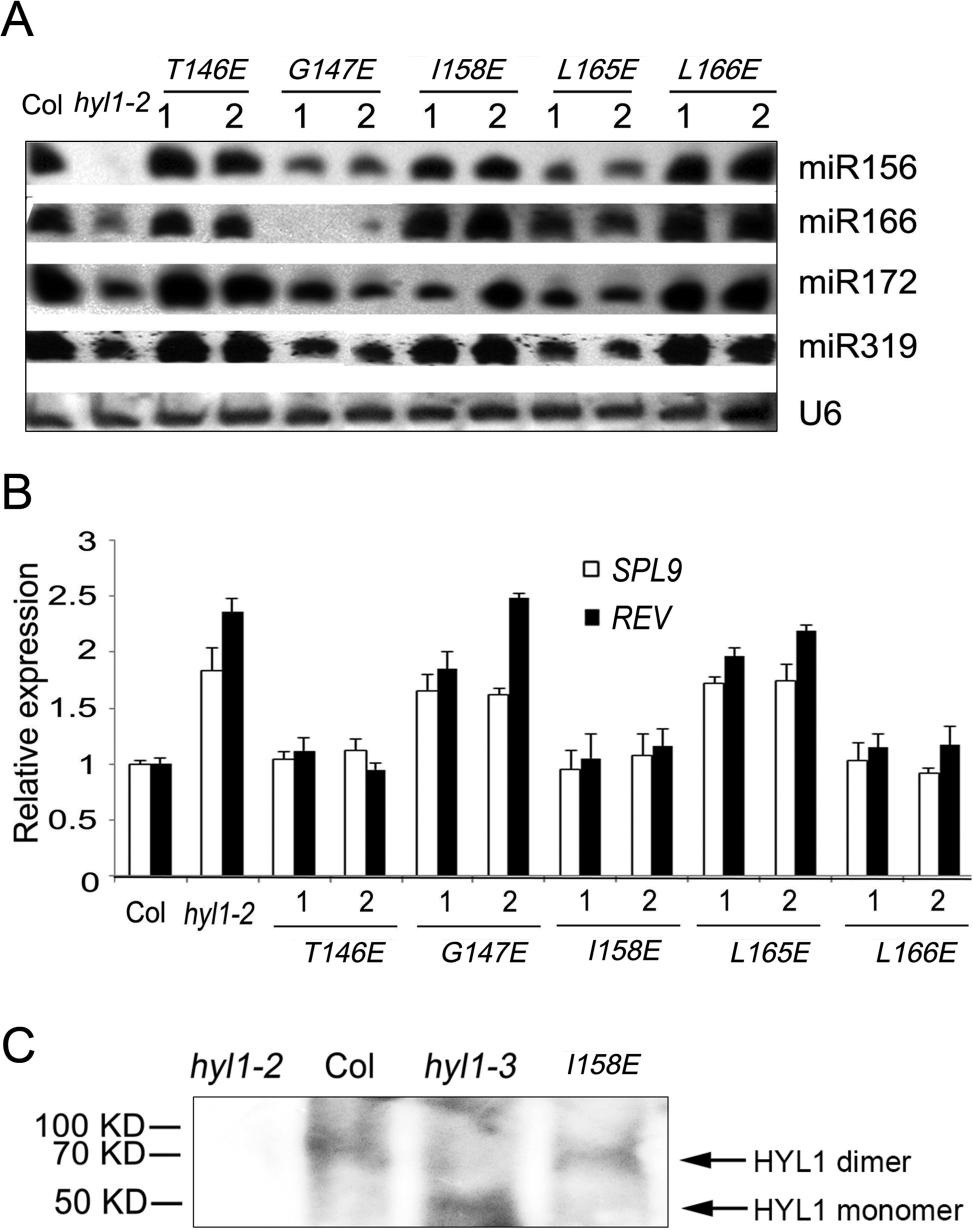
Accumulation of some miRNAs and expression of their targets in the plants deficient in HYL1 homodimerization. (**A**) Northern blot showing miRNA accumulation in the *hyl1-2* mutants and the transgenic plants. (**B**) The expression levels of some miRNA-targeted genes in the *hyl1-2* mutants and the transgenic plants. (**C**) Detection of HYL1 homodimers in the *hyl1-3* mutants. HYL1 antibody was used to detect HYL1 from the total protein extract of the *hyl1-3* plants using the buffer without addition of SDS and urea.

To examine whether the deficiency in HYL1 dimerization affects miRNA biogenesis, we performed northern blotting to detect the accumulation of miRNAs in the *hyl1-2* mutants and transgenic plants with HYL1 mutations. In the *hyl1-2*;*G147E*, *hyl1-2*;*L165E* plants, the accumulation of miR156, miR166, miR172 and miR319 was much less than that in wild-type plants (Figure 5A). By contrast, miR156-targeted *SPL9* and miR165/6-targeted *REV* genes were up-regulated in these plants (Figure 5B). Moreover, the accumulation of these miRNAs in *hyl1-2*;*T146E*, *hyl1-2*;*I158E* and *hyl1-2*;*L166E* was much greater than that in *hyl1-2*, whereas the target genes *SPL9* and *REV* were down-regulated to the levels almost equal to those of the wild-type.

**Figure 6.**
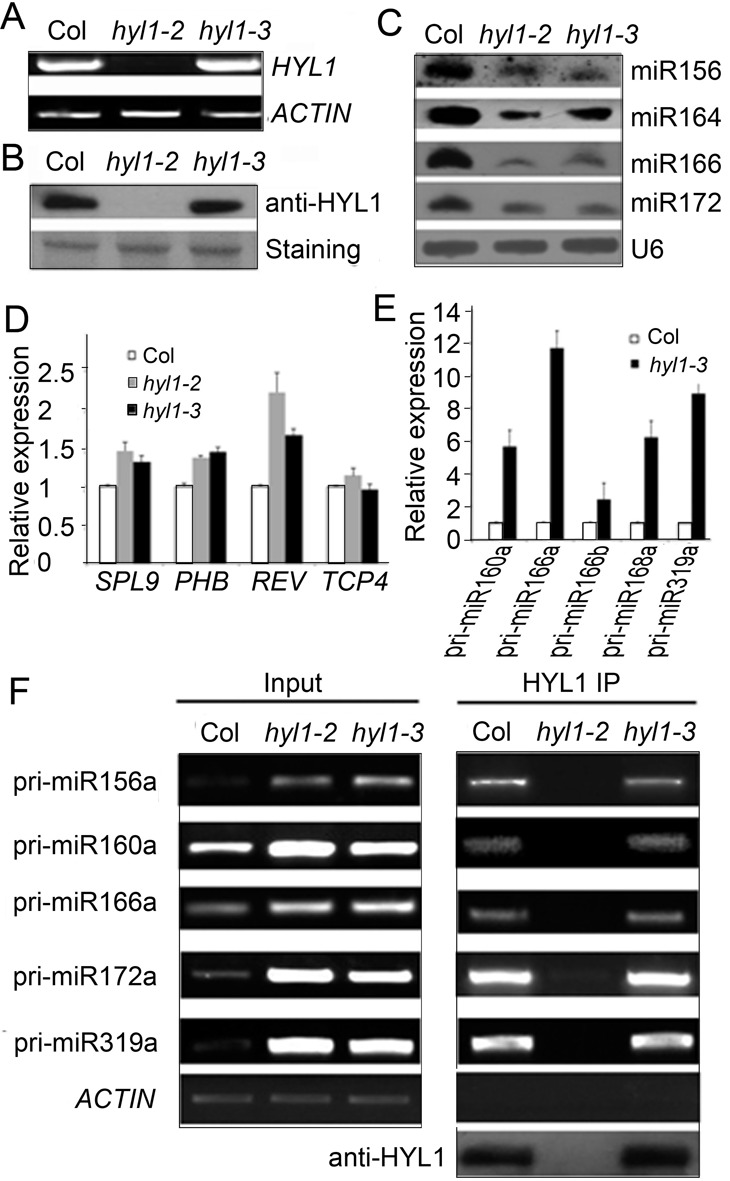
Deficiency of the *hyl1-3* allele in miRNA biogenesis. (**A**) RT-PCR results showing expression of endogenous *G147E* in the *hyl1-3* mutants. (**B**) Western blotting results showing endogenous G147E protein in the *hyl1-3* mutants. (**C**) Northern blotting results showing miRNA accumulation in the *hyl1-3* mutants. (**D**) Real-time PCR results showing the expression levels of some miRNA-targeted genes. (**E**) Real-time PCR results showing the accumulation levels of pri-miRNAs. (**F**) RT-PCR results showing pri-miRNA abundances in immunoprecipitates of Col and *hyl1-3* plants.

To examine whether the endogenous *G147E* in the *hyl1-3* plants influences homodimerization as exogenous *G147E*, we used HYL1 antibody to detect the possible dimerization of G147E in the extracts of *Arabidopsis* under native condition. As expected, we were able to detect HYL1 homodimer in the wild-type and *hyl1-2*;*I158E* plants. (Figure 5C). In the *hyl1-3* plants, HYL1 monomer rather than homodimer was detected. This result indicated that endogenous *G147E* in the *hyl1-3* plants impaired homodimerization *in vivo*. In the *hyl1-3* mutants, the expression levels of *HYL1* gene and HYL1 protein were not different from the wild type (Figure 6A and B); the accumulation of the miRNAs examined was much less than that in wild type, though it was slightly higher than that in *hyl1-2* (Figure 6C), and the genes targeted by these miRNAs were up-regulated (Figure 6D). These results suggest that impairment of HYL1 dimerization affects the biological function of HYL1 in miRNA biogenesis and miRNA-guided gene silencing.

### Dimerization of HYL1 does not affect the accumulation of pri-miRNAs in HYL1/pri-miRNA complexes

To find out how HYL1 regulates the processing of miRNAs through dimerization, we used real-time PCR analysis to examine the accumulation of pri-miRNAs. In the *hyl1-3* seedlings, the expression levels of pri-miR160a, pri-miR166a, pri-miR166b, pri-miR168a and pri-miR319a were much greater than those in the wild-type seedlings (Figure 6E). To exclude the possibility that the endogenous *G147E* affects the transcriptional activities of *MIRNA* genes, we targeted the splicing sites (exon–intron junctions) of *MIR160a*, *MIR166a* and *MIR166b* and detected the levels of their unspliced primary transcripts by qPCR using specific pairs of primers located in the exons and introns (45). The levels of the primary *MIR160a* and *MIR166b* transcripts in *hyl1-3* seedlings were almost the same as those in the wild-type seedlings (Supplementary Figure S6), whereas that of *MIR166a* transcripts were higher than in the wild type. We deduce that the increased accumulation of pri-miR160a and pri-miR166b in the *hyl1-3* mutants is not due to higher transcriptional activity of *MIR160a* and *MIR166b* genes, respectively.

During miRNA processing, HYL1 binds to pri-miRNA. To examine whether the accumulation of pri-miRNAs in HYL1/pri-miRNA complexes is altered with the mutated G147E, we isolated pri-miRNAs cross-linked with HYL1 via immunoprecipitation. RT-PCR analysis of HYL1 immunoprecipitates from the wild-type, *hyl1-2* and *hyl1-3* seedlings indicated that the levels of accumulated pri-miR156a, pri-miR160a, pri-miR166a, pri-miR172a and pri-miR319a in the *hyl1-3* mutants were nearly equal to those in the wild type (Figure 6F). These findings indicate that G147E does not affect the accumulation of pri-miRNAs in HYL1/pri-miRNA complexes.

### HYL1 dimerization enhances the efficiency of miRNA processing

**Figure 7.**
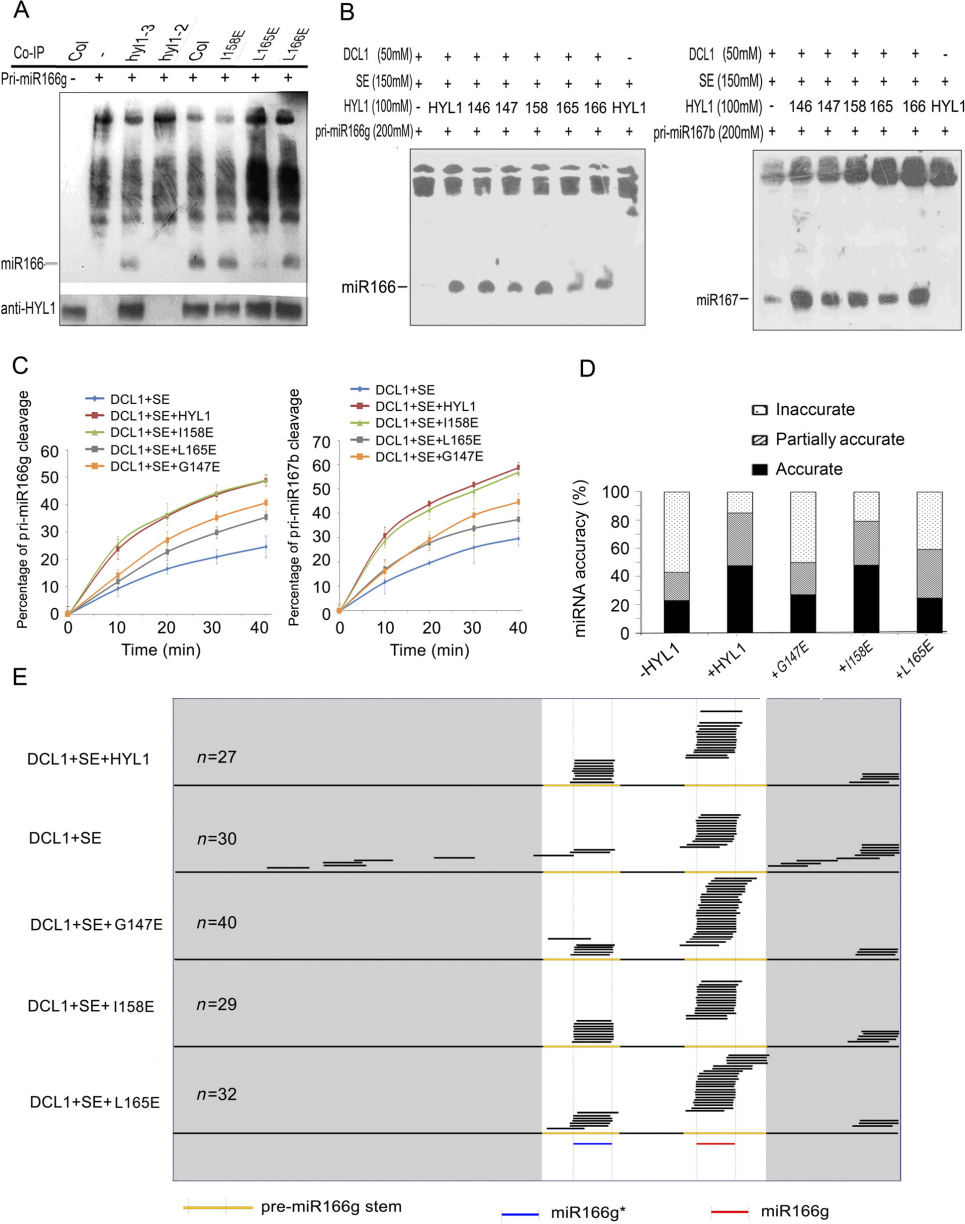
*In vitro* processing of pri-miRNA to mature miRNA. (**A**) Pri-miR166g processing using the immunoprecipitates of the transgenic plants expressing HYL1 mutants. (**B**) *In vitro* pri-miRNA processing using pri-miR166g and pri-miR167b as the substrates. 146, HYL1^T146E^; 147, HYL1^G147E^; 158, HYL1^I158E^; 165, HYL1^L165E^; 166, HYL1^L166E^. (**C**) Pri-miR166g and pri-miR167b processing rates of HYL1 mutants. (**D**) Effects of HYL1 mutants on pri-miR166g processing. −HYL1, absence of HYL1 in the processing complex; +HYL1, HYL1 presence; +G147E, HYL1^G147E^ presence; +I158E, HYL1^I158E^ presence; +L165E, HYL1^L165E^ presence. (**E**) Distribution of the sequenced small RNAs generated from *in vitro* processing reactions of different HYL1 mutants.

To examine the effects of deficiency in HYL1 homodimerization on the efficiency of miRNA processing, we optimized the *in vitro* miRNA processing system (31). HYL1 antibody was applied to immunoprecipitation of the HYL1 complex from the wild-type, *hyl1-2*;*I158E*, *hyl1-2*;*L165E*, *hyl1-2*;*L166E* and *hyl1-3* plants. Addition of *in vitro* transcribed pri-miR166g to aliquots of the protein extracts yielded mature miR166 for all plants except the *hyl1-2* mutant (Figure 7A). Northern blotting showed that the accumulation of miR166 in *hyl1-2*;*I158E* and *hyl1-2*;*L166E* was similar to that in the wild type, whereas that in the *hyl1-2*;*L165E* and *hyl1-3* plants was much lower. These results suggest that a deficiency in HYL1 homodimerization reduces the efficiency of pri-miRNA processing.

Meanwhile, *in vitro* miRNA processing was performed to optimize the components of the dicing complex using puriﬁed recombinant HYL1, DCL1 and SE (Supplementary Figure S5A). *In vitro* transcribed and puriﬁed pri-miR166g substrates were added to reaction solutions to examine the activities of the recombinant proteins. Under standard conditions described previously (19), DCL1 alone processed the pri-miR166g into mature miR166, whereas HYL1 and SE enhanced the cleavage of pri-miR166g (Supplementary Figure S5B). However, when HYL1 was replaced by an equal amount of each HYL1 mutant in the processing system (Figure 7B), the G147E and L165E mutants generated less miR166 and miR167 from the pri-miR166g or pri-miR167b substrates, respectively, than did the wild-type HYL1 and I158E samples. A kinetic analysis was performed to test the processing rate by quantitation of the remnant pri-miR166g and pri-miR167b substrates with real-time PCR. In the processing of both pri-miR166g and pri-miR167b, turnover of the substrates was remarkably reduced by G147E and L165E compared with wild-type HYL1 (Figure 7C). These findings strongly support our conclusion that HYL1 dimerization plays a role in the efficiency of pri-miRNA processing.

### HYL1 dimerization is necessary for miRNA accuracy

HYL1 has been reported to enhance the accuracy of mature miRNAs (19), and thus, we examined whether the miRNA accuracy is regulated by HYL1 dimerization within *in vitro* miRNA processing system. Equal amounts of the individual HYL1 mutants were added to each miRNA processing system, which was then incubated with DCL1, SE and pri-miR166g. The small RNAs generated from the pri-miRNAs were cloned, and the resultant small RNA libraries were constructed for sequencing. According to the extent of miRNA accuracy, small RNAs were assigned to three classes: accurate (mature miR166 or miR166*), partially accurate (position shifting by 2 nt or less) and inaccurate (position shifting by more than 2 nt).

Effects of HYL1 absence on miRNA accuracy were obvious. The inaccuracy of miR166 for DCL1+SE complexes was 56% (Figure 7D), which is four times higher than that for DCL1+HYL1+SE complexes. A large proportion of inaccurate miR166 for DCL1+SE complexes were derived from the regions beyond the stem of pre-miRNA (Figure 7E), in contrast with those for DCL1+HYL1+SE complexes in that they were derived from pre-miRNA regions. Like the wild-type HYL1, I158E showed low inaccuracy in miR166 and miR166*. By contrast, G147E and L165E displayed greater inaccuracy than the wild-type HYL1, and their inaccuracy was elevated according to the degree at which HYL1 was absent. The inaccurate small RNAs arising from DCL1+SE+G147E and DCL1+SE+L165E complexes were found mainly in regions that overlapped with miR166 and miR166*. These results reveal that HYL1 homodimers are required for miRNA accuracy because they prevent the production of inaccurate small RNAs.

### HYL1 homodimers contribute to correct selection of pri-miRNA cleavage sites by DCL1

**Figure 8.**
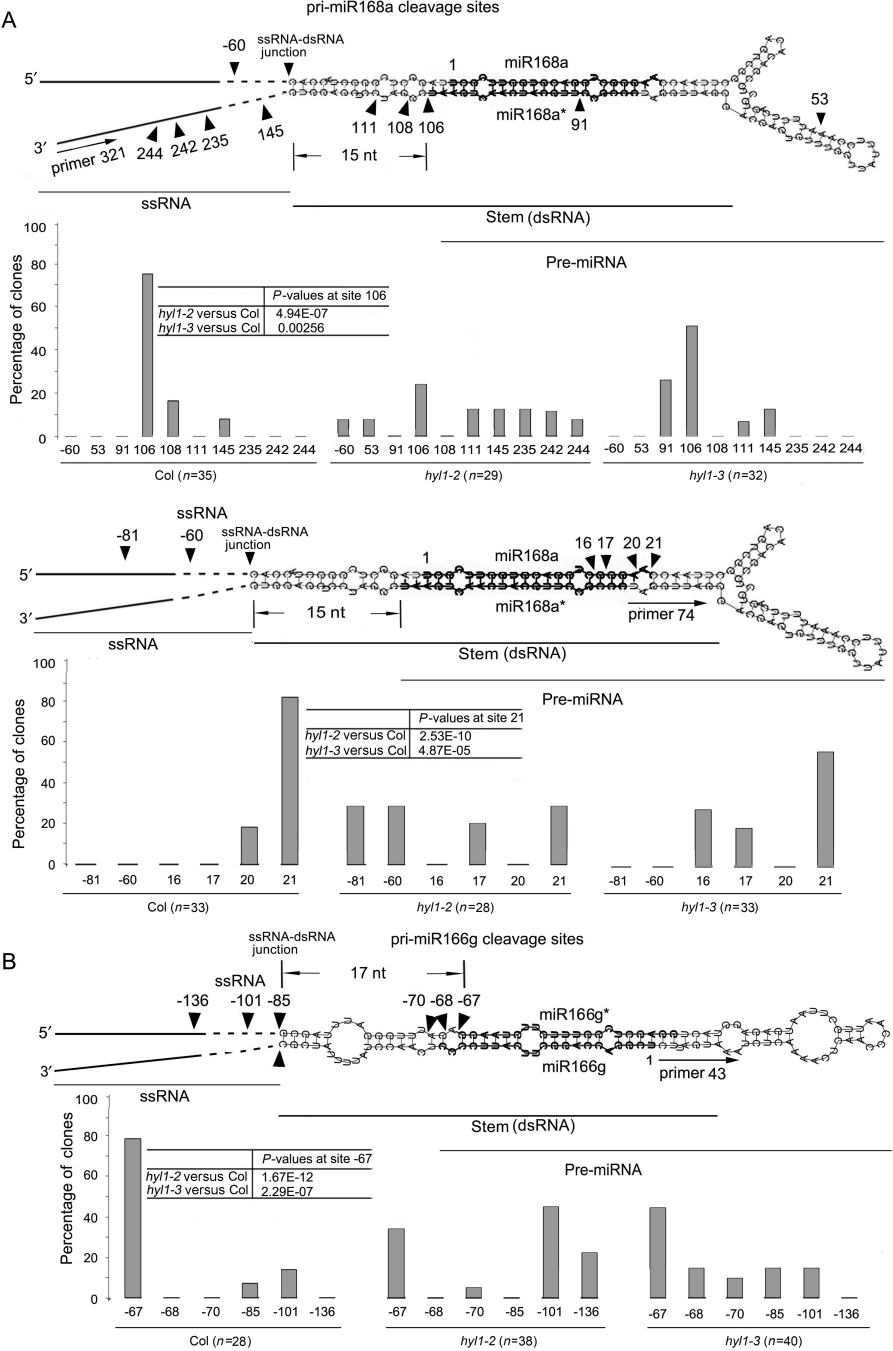
Correct selection of pri-miRNA cleavage sites *in vivo*. (**A**) Cleavage sites on pri-miR168a using primer 321 (an internal primer of the two sequence-specific primers upstream of miR168a*) and primer 74 (an internal primer of the two sequence-specific primers downstream of miR168a*). (**B**) Cleavage sites on pri-miR166g using primer 43 (an internal primer of the two sequence-specific primers upstream of miR166). Arrowheads indicate cleavage sites, and numbers above or below arrowheads list the number of nucleotides between the sites and the first nucleotides of mature miRNA. The numbers in open boxes are the percentages of correct and incorrect cleavage sites. Pearson's chi-squared test (*χ*^2^) is applied to percentages of correct cleavage sites to evaluate significance of difference between the wild-type, *hyl1-2* and *hyl1-3* plants.

To understand how HYL1 homodimerization affects pri-miRNA cleavage we used 5′ RACE PCR analysis to detect the cleavage sites *in vivo*. In wild-type plants, primers 321 and 43 generated the correct cleavage sites at the ends of miRNA* regions on pri-miR168a and pri-miR166g, respectively (Figure 8A and B). In the *hyl1-2* and *hyl1-3* plants, percentages of the correct cleavage sites generated with these primers were much lower than that of wild-type plants. These results show that disruption of HYL1 homodimerization impairs the correct selection of pri-miRNA cleavage sites.

Aberrance of cleavage sites in pri-miRNAs cause the change in sequence of pre-miRNAs, probably leading to the production of inaccurate miRNAs. To detect the cleavage sites at the 3′ ends of miRNA regions, we designed other primers specific for pri-miR168a. Primer 74 generated the correct cleavage sites at the 3′ ends of miR168 regions in the wild-type plants. In *hyl1-2* and *hyl1-3* plants, percentages of the correct cleavage sites with this primer were much lower than that of wild-type plants, corresponding to the significant increase in percentages of the incorrect cleavage sites. This result reveals that disruption of HYL1 dimerization impairs the correct cleavage site selection at the 3′ ends of miRNA regions.

The secondary structure of a pri-miRNA consists of four regions: 5′ and 3′ single-stranded RNAs (ssRNAs), double-stranded RNA (dsRNA; a stem bearing mature miRNA and miRNA*) and loop. There is a junction between ssRNA and dsRNA. In wild-type plants, primers 321 and 43 generated the correct cleavage sites 15 and 17 nt away from the ssRNA–dsRNA junction, respectively, while primer 74 did 15+21 nt away from the junction (Figure 8A and B). By contrast, these primers produced many incorrect cleavage sites in the *hyl1-2* and *hyl1-3* mutants. The incorrect cleavage sites of *hyl1-2* plants were mainly on the stem and in regions outside the stem, whereas those of the *hyl1-3* plants were mainly near the 5′ and 3′ ends of miRNA regions with different distances (1–5 nt shift). These findings reveal that disruption of HYL1 homodimerization increases the incorrect cleavage sites mainly in the dsRNA regions, resulting in the nucleotide shift near the 5′ and 3′ ends of miRNA regions.

## DISCUSSION

### HYL1 functions through homodimerization

miRNA accuracy is an important factor in miRNA-directed gene silencing. HYL1 has been considered to play an essential role in miRNA accuracy, and we wondered whether some novel proteins help HYL1 accomplish this role. Yeast two-hybrid screening revealed that HYL1 functions in the form of a dimer in miRNA processing. *In vitro* pull-down assays confirmed the homodimerization of HYL1. From the crystal structure of HYL1, Yang *et al.* (35) proposed that dsRBD2 harbors a putative dimerization interface. This dimerization interface is primarily located between the β1 strand of one HYL1 dsRBD2 molecule and the β3′ strand of its symmetric related molecule in a parallel interaction mode. In another work, however, dsRBD2 behaves exclusively as a monomer in solution (46). We found that HYL1 proteins form homodimers through the dsRBD2 domains. This finding is consistent with the results of Yang *et al.* (35). In their work, eight triple mutants with point mutations in the dsRBD2 region yielded misfolded proteins or no protein expression at all. We identified two point mutations that disrupted dimerization. Gly^147^ and Leu^165^ are critical for HYL1 homodimerization. In our BiFC experiments, the homodimerization of G147E/HYL1 or L165E/HYL1 was hardly detectable compared with that of HYL1/HYL1. In pull-down assays, however, the homodimerization of G147E/HYL1 or L165E/HYL1 was relatively weaker than that of HYL1/HYL1. This indicates that the strength of HYL1 homodimerization with its mutants is different under the *in vivo* and *in vitro* conditions.

In animals, Drosha and DGCR8 form a complex termed the Microprocessor to cleave the pri-miRNAs into pre-miRNAs (37–39). The RNA-binding protein DGCR8 is a heme-binding protein and the association with heme promotes dimerization of DGCR8 (47,48). The heme-bound DGCR8 dimer seems to be more active in triggering pri-miRNA cleavage *in vitro*. Unfortunately, homodimerization of DGCR8 had not been identified *in vivo*, and its putative role in pri-miRNA processing not defined. Interestingly, HYL1 proteins are able to form homodimers in plants. This reminds us that HYL1 may have the same function as DGCR8 in pri-miRNA processing.

### HYL1 homodimers are crucial for correct selection of cleavage sites in pri-miRNA

HYL1-directed miRNA biogenesis is generally divided into five biological steps: (i) interaction of HYL1 with DCL1 and SE, (ii) binding of HYL1 to pri-miRNA, (iii) correct selection of pri-miRNA cleavage sites, (iv) efficient processing of pri-miRNA and (v) maturation of accurate miRNA. The absence of HYL1 in the *hyl1-2* mutants actually affects all of these steps because the D-body is aberrant. Unlike *hyl1-2* mutants, however, disruption of HYL1 homodimerization in the *hyl1-3* allele (or *G147E* and *L165E* mutants) causes the incorrect selection of pri-miRNA cleavage sites. This disruption neither interrupts the interaction of HYL1 with DCL1 and SE nor decreases HYL1 binding affinity to pri-miRNA. In this sense, the correct selection of cleavage sites is critical for miRNA accuracy.

Exogenous G147E and L165E generated lower levels of miRNAs than the wild-type HYL1, and endogenous G147E in the *hyl1-3* plants led to deficient miRNA biogenesis as well. Either endogenous or exogenous G147E causes the mutant phenotypes because of miRNA inaccuracy. The importance of Gly^147^ and Leu^165^ for plant phenotype reveals that HYL1 homodimerization is essential for miRNA accuracy and plant development.

Pri-miRNA processing is an early and critical stage of miRNA biogenesis as it defines the sequence of pre-miRNAs and mature miRNAs by generating one end of the molecule. In this study, the strand-specific primers uncovered the first cleavage sites of pri-miR168a and pri-miR166g at the 3′ ends of miR168* and miR166, respectively. In animal cells, a critical step in human miRNA maturation is the processing of pri-miRNA transcripts by the nuclear RNaseIII enzyme Drosha to generate pre-miRNA (7,49). Drosha selectively cleaves RNA hairpins bearing a large terminal loop, with cleavage sites largely determined by distance along the RNA (8). In addition, LOQS can change the cleavage sites targeted by Dicer, leading to the production of miRNAs with target specificities different from those generated by Dicer alone or Dicer bound to other protein partners (40).

### HYL1 homodimers ensure the correct distance from ssRNA–dsRNA junction in pri-miRNA

Aberrance of cleavage site selection in pri-miRNAs can cause changes in the length and/or sequences of pre-miRNAs, leading to the inaccuracy in miRNAs. In animal cells, Drosha–DGCR8 complex cleaves the pri-miRNAs at the sites ∼11 bp away from the ssRNA–dsRNA junction (50). In plants, an imperfectly paired stem of ∼15 bp below the miRNA:miRNA* duplex is considered to be a key element for the initial pri-miRNA cleavage (51,52). The secondary structure of pri-miRNAs influences the recognition and cleavage pattern during their processing (53). Recently, one report mentioned that DCL1 complexes cleaved pri-miRNAs at 16–17 bp away from reference ssRNA–dsRNA junctions for either canonical or noncanonical processing (54). Thus, a ‘distance mechanism’ seems to ensure the correct selection of cleavage sites on pri-miRNAs. Our results provide evidence that the correct cleavage at the 5′ end of miRNA region in wild-type plants occurs just 15 or 17 nt away from the ssRNA–dsRNA junction. In the *hyl1-3* mutants, the correct cleavage sites at the ends of miRNA regions decrease whereas the incorrect ones in dsRNA regions increase. By contrast, a large proportion of the incorrect cleavage sites in the *hyl1-2* plants exist in the ssRNA regions, meaning that the absence of HYL1 increase the incorrect cleavage sites in ssRNA regions. This fact suggests that the distance of cleavage sites from ssRNA–dsRNA junction depends on dimerization of HYL1. Possibly, HYL1 homodimers act as a ruler to recognize the ssRNA–dsRNA junction and to direct DCL1 to cleave 15–17 nt away from the junction.

**Figure 9.**
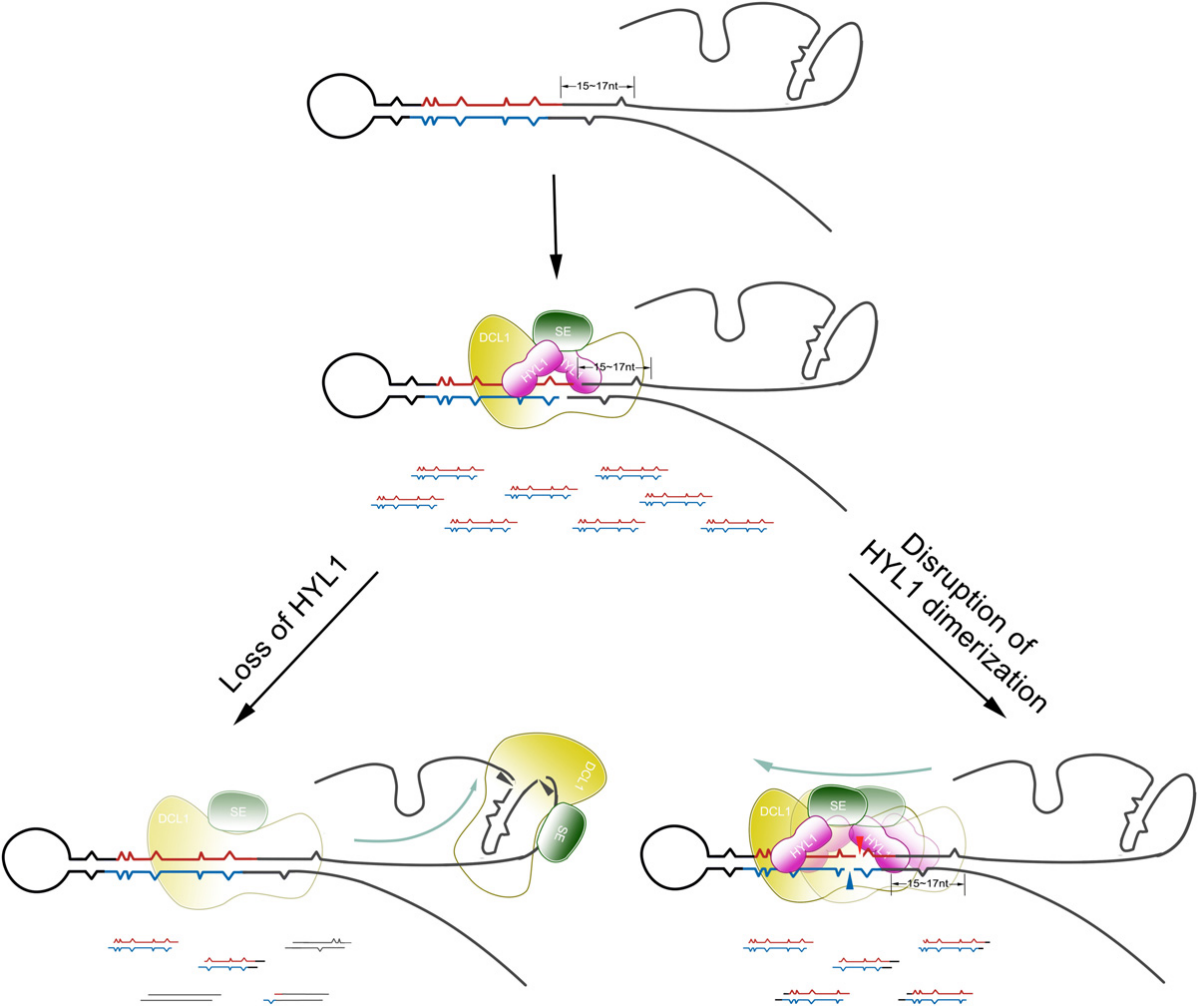
Model of how correct selection of pri-miRNA cleavage sites is affected by the absence of HYL1 and disruption of HYL1 dimerization. HYL1 promotes the correct selection of pri-miRNA cleavage sites through interaction with DCL1 and SE. The absence of HYL1 causes the incorrect selection of pri-miRNA cleavage sites mainly in the ssRNA regions, while disruption of HYL1 homodimerization results in the incorrect selection of pri-miRNA cleavage sites mainly in stem because pri-miRNA cleavage sites are incorrectly chosen. Wavy lines in red and blue indicate accurate miRNAs and miRNAs*, respectively. The lines in black show the incorrect ones, and the lines in mosaic red and black display the partially accurate ones.

The model supposed in Figure 9 suggests that disruption of HYL1 homodimerization impairs the correct selection of cleavage sites in pri-miRNA because the distance from ssRNA–dsRNA junction is altered. The mechanisms by which the correct selection of cleavage sites occurs in pri- and/or pre-miRNA have remained a mystery. Nevertheless, our results indicate that this process may be dependent on HYL1 binding to the correct sites on pri-miRNA or pre-miRNAs. Further study on the binding sites of HYL1 on pri-miRNAs and pre-miRNAs will facilitate an improved understanding of the molecular mechanisms underlying miRNA biogenesis.

## SUPPLEMENTARY DATA

Supplementary Data are available at NAR Online.

SUPPLEMENTARY DATA

## References

[1] Bartel D.P. (2004). MicroRNAs: genomics, biogenesis, mechanism, and function. Cell.

[2] Carrington J.C., Ambros V. (2003). Role of microRNAs in plant and animal development. Science.

[3] Nam J.W., Rissland O.S., Koppstein D., Abreu-Goodger C., Jan C.H., Agarwal V., Yildirim M.A., Rodriguez A., Bartel D.P. (2014). Global analyses of the effect of different cellular contexts on microRNA targeting. Mol. Cell.

[4] Croce C.M., Calin G.A. (2005). miRNAs, cancer, and stem cell division. Cell.

[5] Li S., Liu J., Liu Z., Li X., Wu F., He Y. (2014). HEAT-INDUCED TAS1 TARGET1 mediates thermotolerance via HEAT STRESS TRANSCRIPTION FACTOR A1a-directed pathways in Arabidopsis. Plant Cell.

[6] Palatnik J.F., Allen E., Wu X., Schommer C., Schwab R., Carrington J.C., Weigel D. (2003). Control of leaf morphogenesis by microRNAs. Nature.

[7] Lee Y., Ahn C., Han J., Choi H., Kim J., Yim J., Lee J., Provost P., Radmark O., Kim S. (2003). The nuclear RNase III Drosha initiates microRNA processing. Nature.

[8] Zeng Y., Cullen B.R. (2005). Efficient processing of primary microRNA hairpins by Drosha requires flanking nonstructured RNA sequences. J. Biol. Chem..

[9] Lund E., Guttinger S., Calado A., Dahlberg J.E., Kutay U. (2004). Nuclear export of microRNA precursors. Science.

[10] Yi R., Qin Y., Macara I.G., Cullen B.R. (2003). Exportin-5 mediates the nuclear export of pre-microRNAs and short hairpin RNAs. Genes Dev..

[11] Bernstein E., Caudy A.A., Hammond S.M., Hannon G.J. (2001). Role for a bidentate ribonuclease in the initiation step of RNA interference. Nature.

[12] Kurihara Y., Watanabe Y. (2004). Arabidopsis micro-RNA biogenesis through Dicer-like 1 protein functions. Proc. Natl. Acad. Sci. U.S.A..

[13] Tang G., Reinhart B.J., Bartel D.P., Zamore P.D. (2003). A biochemical framework for RNA silencing in plants. Genes Dev..

[14] Brodersen P., Sakvarelidze-Achard L., Bruun-Rasmussen M., Dunoyer P., Yamamoto Y.Y., Sieburth L., Voinnet O. (2008). Widespread translational inhibition by plant miRNAs and siRNAs. Science.

[15] Baumberger N., Baulcombe D.C. (2005). Arabidopsis ARGONAUTE1 is an RNA slicer that selectively recruits microRNAs and short interfering RNAs. Proc. Natl. Acad. Sci. U.S.A..

[16] Lanet E., Delannoy E., Sormani R., Floris M., Brodersen P., Crete P., Voinnet O., Robaglia C. (2009). Biochemical evidence for translational repression by Arabidopsis microRNAs. Plant Cell.

[17] Li S., Liu L., Zhuang X., Yu Y., Liu X., Cui X., Ji L., Pan Z., Cao X., Mo B. (2013). MicroRNAs inhibit the translation of target mRNAs on the endoplasmic reticulum in Arabidopsis. Cell.

[18] Llave C., Xie Z., Kasschau K.D., Carrington J.C. (2002). Cleavage of Scarecrow-like mRNA targets directed by a class of Arabidopsis miRNA. Science.

[19] Dong Z., Han M.H., Fedoroff N. (2008). The RNA-binding proteins HYL1 and SE promote accurate in vitro processing of pri-miRNA by DCL1. Proc. Natl. Acad. Sci. U.S.A..

[20] Grigg S.P., Canales C., Hay A., Tsiantis M. (2005). SERRATE coordinates shoot meristem function and leaf axial patterning in Arabidopsis. Nature.

[21] Han M.H., Goud S., Song L., Fedoroff N. (2004). The Arabidopsis double-stranded RNA-binding protein HYL1 plays a role in microRNA-mediated gene regulation. Proc. Natl. Acad. Sci. U.S.A..

[22] Lobbes D., Rallapalli G., Schmidt D.D., Martin C., Clarke J. (2006). SERRATE: a new player on the plant microRNA scene. EMBO Rep..

[23] Fang Y., Spector D.L. (2007). Identification of nuclear dicing bodies containing proteins for microRNA biogenesis in living Arabidopsis plants. Curr. Biol..

[24] Liu C., Axtell M.J., Fedoroff N.V. (2012). The helicase and RNaseIIIa domains of Arabidopsis Dicer-Like1 modulate catalytic parameters during microRNA biogenesis. Plant Physiol..

[25] Yu B., Bi L., Zheng B., Ji L., Chevalier D., Agarwal M., Ramachandran V., Li W., Lagrange T., Walker J.C. (2008). The FHA domain proteins DAWDLE in Arabidopsis and SNIP1 in humans act in small RNA biogenesis. Proc. Natl. Acad. Sci. U.S.A..

[26] Kim S., Yang J.Y., Xu J., Jang I.C., Prigge M.J., Chua N.H. (2008). Two cap-binding proteins CBP20 and CBP80 are involved in processing primary MicroRNAs. Plant Cell Physiol..

[27] Yang Z., Ebright Y.W., Yu B., Chen X. (2006). HEN1 recognizes 21–24 nt small RNA duplexes and deposits a methyl group onto the 2′ OH of the 3′ terminal nucleotide. Nucleic Acids Res..

[28] Wu X., Shi Y., Li J., Xu L., Fang Y., Li X., Qi Y. (2013). A role for the RNA-binding protein MOS2 in microRNA maturation in Arabidopsis. Cell Res..

[29] Manavella P.A., Hagmann J., Ott F., Laubinger S., Franz M., Macek B., Weigel D. (2012). Fast-forward genetics identifies plant CPL phosphatases as regulators of miRNA processing factor HYL1. Cell.

[30] Lu C., Fedoroff N. (2000). A mutation in the Arabidopsis HYL1 gene encoding a dsRNA binding protein affects responses to abscisic acid, auxin, and cytokinin. Plant Cell.

[31] Wu F., Yu L., Cao W., Mao Y., Liu Z., He Y. (2007). The N-terminal double-stranded RNA binding domains of Arabidopsis HYPONASTIC LEAVES1 are sufficient for pre-microRNA processing. Plant Cell.

[32] Li S., Yang X., Wu F., He Y. (2012). HYL1 controls the miR156-mediated juvenile phase of vegetative growth. J. Exp. Bot..

[33] Lian H., Li X., Liu Z., He Y. (2013). HYL1 is required for establishment of stamen architecture with four microsporangia in Arabidopsis. J. Exp. Bot..

[34] Liu Z., Jia L., Wang H., He Y. (2011). HYL1 regulates the balance between adaxial and abaxial identity for leaf flattening via miRNA-mediated pathways. J.Exp. Bot..

[35] Yang S.W., Chen H.Y., Yang J., Machida S., Chua N.H., Yuan Y.A. (2010). Structure of Arabidopsis HYPONASTIC LEAVES1 and its molecular implications for miRNA processing. Structure.

[36] Denli A.M., Tops B.B., Plasterk R.H., Ketting R.F., Hannon G.J. (2004). Processing of primary microRNAs by the Microprocessor complex. Nature.

[37] Gregory R.I., Yan K.P., Amuthan G., Chendrimada T., Doratotaj B., Cooch N., Shiekhattar R. (2004). The Microprocessor complex mediates the genesis of microRNAs. Nature.

[38] Han J., Lee Y., Yeom K.H., Kim Y.K., Jin H., Kim V.N. (2004). The Drosha-DGCR8 complex in primary microRNA processing. Genes Dev..

[39] Landthaler M., Yalcin A., Tuschl T. (2004). The human DiGeorge syndrome critical region gene 8 and Its D. melanogaster homolog are required for miRNA biogenesis. Curr.Biol..

[40] Fukunaga R., Han B.W., Hung J.H., Xu J., Weng Z., Zamore P.D. (2012). Dicer partner proteins tune the length of mature miRNAs in flies and mammals. Cell.

[41] Lee H.Y., Doudna J.A. (2012). TRBP alters human precursor microRNA processing in vitro. RNA.

[42] MacRae I.J., Ma E., Zhou M., Robinson C.V., Doudna J.A. (2008). In vitro reconstitution of the human RISC-loading complex. Proc. Natl. Acad. Sci. U.S.A..

[43] Haase A.D., Jaskiewicz L., Zhang H., Laine S., Sack R., Gatignol A., Filipowicz W. (2005). TRBP, a regulator of cellular PKR and HIV-1 virus expression, interacts with Dicer and functions in RNA silencing. EMBO Rep..

[44] Kurihara Y., Takashi Y., Watanabe Y. (2006). The interaction between DCL1 and HYL1 is important for efficient and precise processing of pri-miRNA in plant microRNA biogenesis. RNA.

[45] Liu F., Quesada V., Crevillen P., Baurle I., Swiezewski S., Dean C. (2007). The Arabidopsis RNA-binding protein FCA requires a lysine-specific demethylase 1 homolog to downregulate FLC. Molecular Cell.

[46] Rasia R.M., Mateos J., Bologna N.G., Burdisso P., Imbert L., Palatnik J.F., Boisbouvier J. (2010). Structure and RNA interactions of the plant MicroRNA processing-associated protein HYL1. Biochemistry.

[47] Faller M., Matsunaga M., Yin S., Loo J.A., Guo F. (2007). Heme is involved in microRNA processing. Nat. Struct. Mol. Biol..

[48] Senturia R., Faller M., Yin S., Loo J.A., Cascio D., Sawaya M.R., Hwang D., Clubb R.T., Guo F. (2010). Structure of the dimerization domain of DiGeorge critical region 8. Protein Sci..

[49] Lee Y., Jeon K., Lee J.T., Kim S., Kim V.N. (2002). MicroRNA maturation: stepwise processing and subcellular localization. EMBO. J..

[50] Han J., Lee Y., Yeom K.H., Nam J.W., Heo I., Rhee J.K., Sohn S.Y., Cho Y., Zhang B.T., Kim V.N. (2006). Molecular basis for the recognition of primary microRNAs by the Drosha-DGCR8 complex. Cell.

[51] Song L., Axtell M.J., Fedoroff N.V. (2010). RNA secondary structural determinants of miRNA precursor processing in Arabidopsis. Curr. Biol..

[52] Werner S., Wollmann H., Schneeberger K., Weigel D. (2010). Structure determinants for accurate processing of miR172a in Arabidopsis thaliana. Curr. Biol..

[53] Bologna N.G., Schapire A.L., Zhai J., Chorostecki U., Boisbouvier J., Meyers B.C., Palatnik J.F. (2013). Multiple RNA recognition patterns during microRNA biogenesis in plants. Genome Res..

[54] Zhu H., Zhou Y., Castillo-Gonzalez C., Lu A., Ge C., Zhao Y.T., Duan L., Li Z., Axtell M.J., Wang X.J. (2013). Bidirectional processing of pri-miRNAs with branched terminal loops by Arabidopsis Dicer-like1. Nat. Struct. Mol. Biol..

