# Supplementary material for: Homodimerization of HYL1 ensures the correct selection of cleavage sites in primary miRNA
Source: Nucleic Acids Res. 2014 Oct 7;42(19):12224–36. doi: 10.1093/nar/gku907 (PMC4231765; doi:10.1093/nar/gku907)
Supplement: SUPPLEMENTARY DATA [file supp_42_19_12224__index.html]

Homodimerization of HYL1 ensures the correct selection of cleavage sites in primary miRNA — SUPPLEMENTARY DATA 

# Homodimerization of HYL1 ensures the correct selection of cleavage sites in primary miRNA

## SUPPLEMENTARY DATA

**Files in this Data Supplement:**

- SUPPLEMENTARY DATA
