# Supplementary material for: Homodimerization of HYL1 ensures the correct selection of cleavage sites in primary miRNA
Source: Nucleic Acids Res. 2014 Oct 7;42(19):12224–36. doi: 10.1093/nar/gku907 (PMC4231765; doi:10.1093/nar/gku907)
Supplement: SUPPLEMENTARY DATA [file supp_gku907_nar-01706-y-2014-File011.docx]

**Supplemental Information**


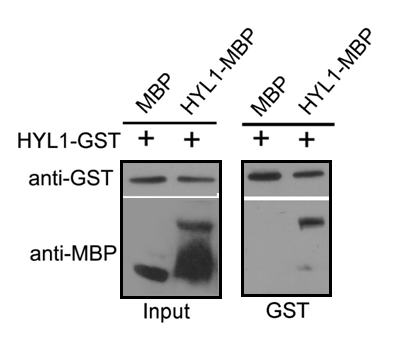


Figure S1. Pull-down assay results for HYL1 homodimers. MBP-bait protein itself is served as negative control.


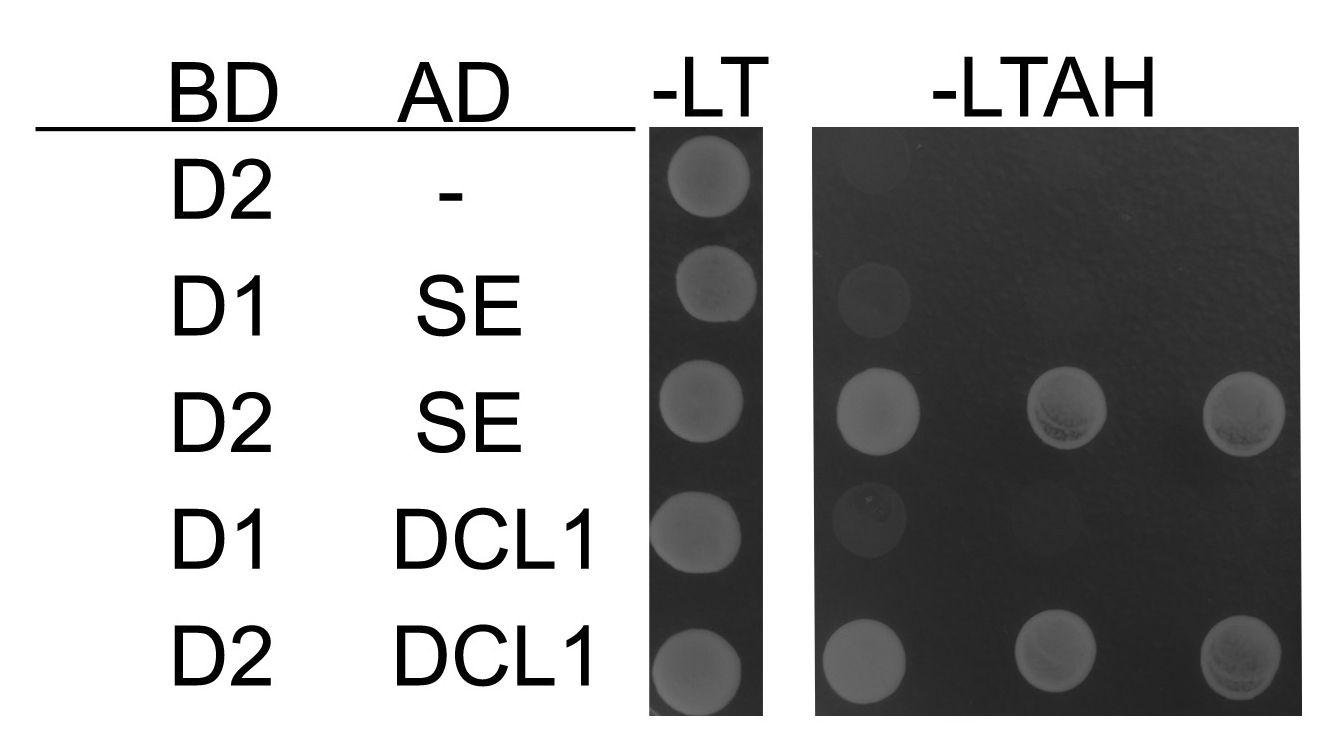


Figure S2. Interaction between HYL1 variants and DCL1 and SE in yeast-two-hybrid analysis.


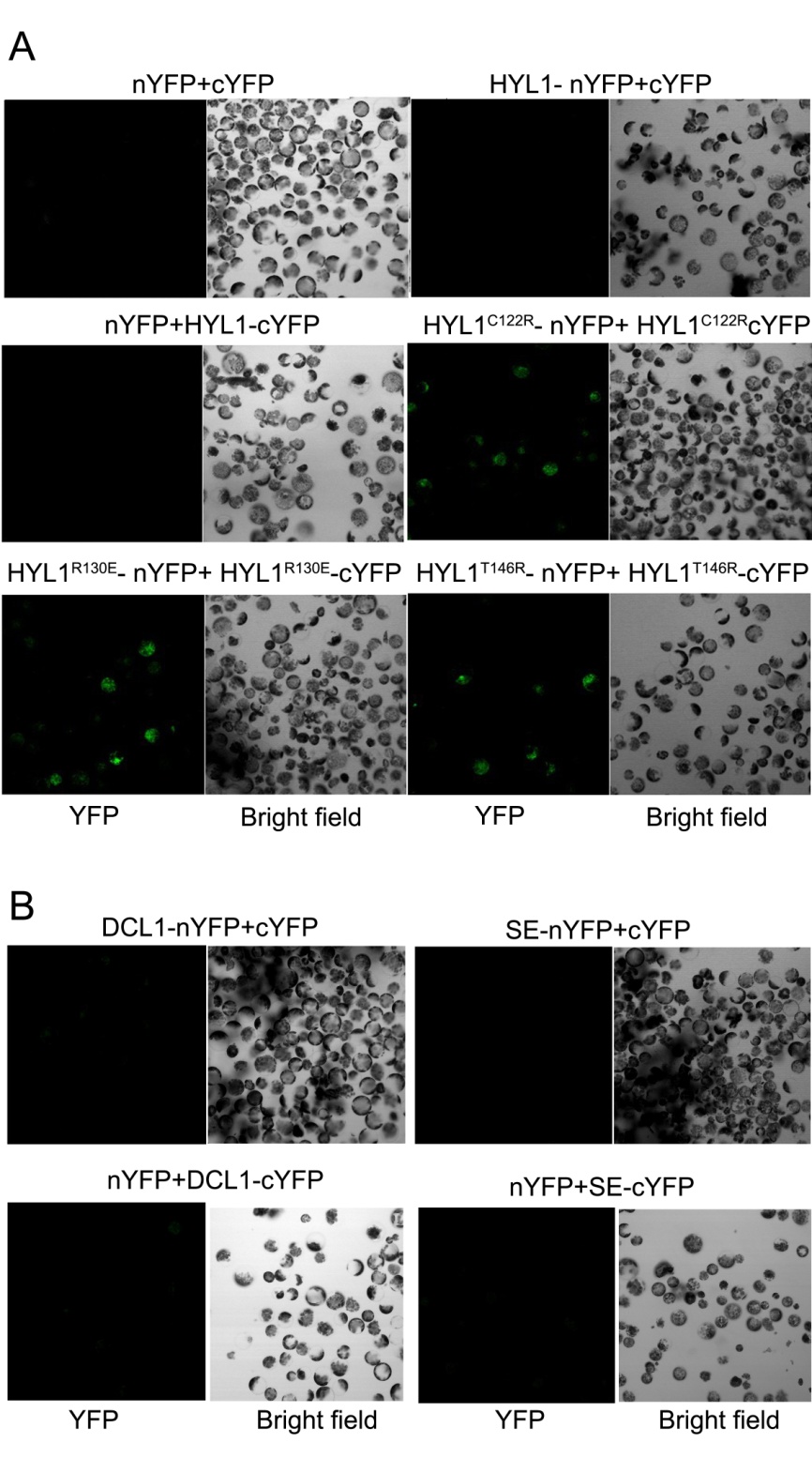


Figure S3. Fluorescence of protoplasts showing protein-protein interaction of some HYL1 mutants with themselves in BiFC analysis.

1. Some HYL1 mutants with themselves.
2. Negative controls for interaction with DCL1 and SE.


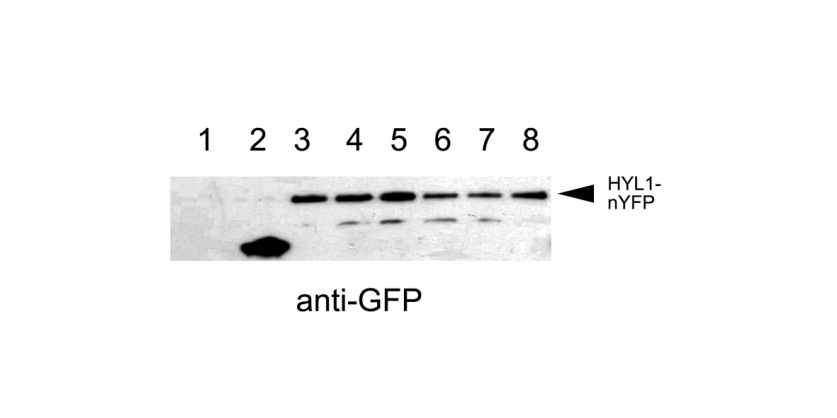


Figure S4. Protein levels of HYL1 isoforms in protoplasts with transient expression of HYL1 isoforms.

Lane 1: protoplasts without any transformation. Lane 2: nYFP/cYFP.

Lane 3: HYL1-nYFP/HYL1-cYFP. Lane 4: T146E-nYFP/T146E-cYFP. Lane 5: G147E-nYFP/G147E-cYFP. Lane 6: I158E-nYFP/I158E-cYFP. Lane 7: L165E-nYFP/L165E-cYFP. Lane 8: L166E-nYFP/L166E-cYFP.


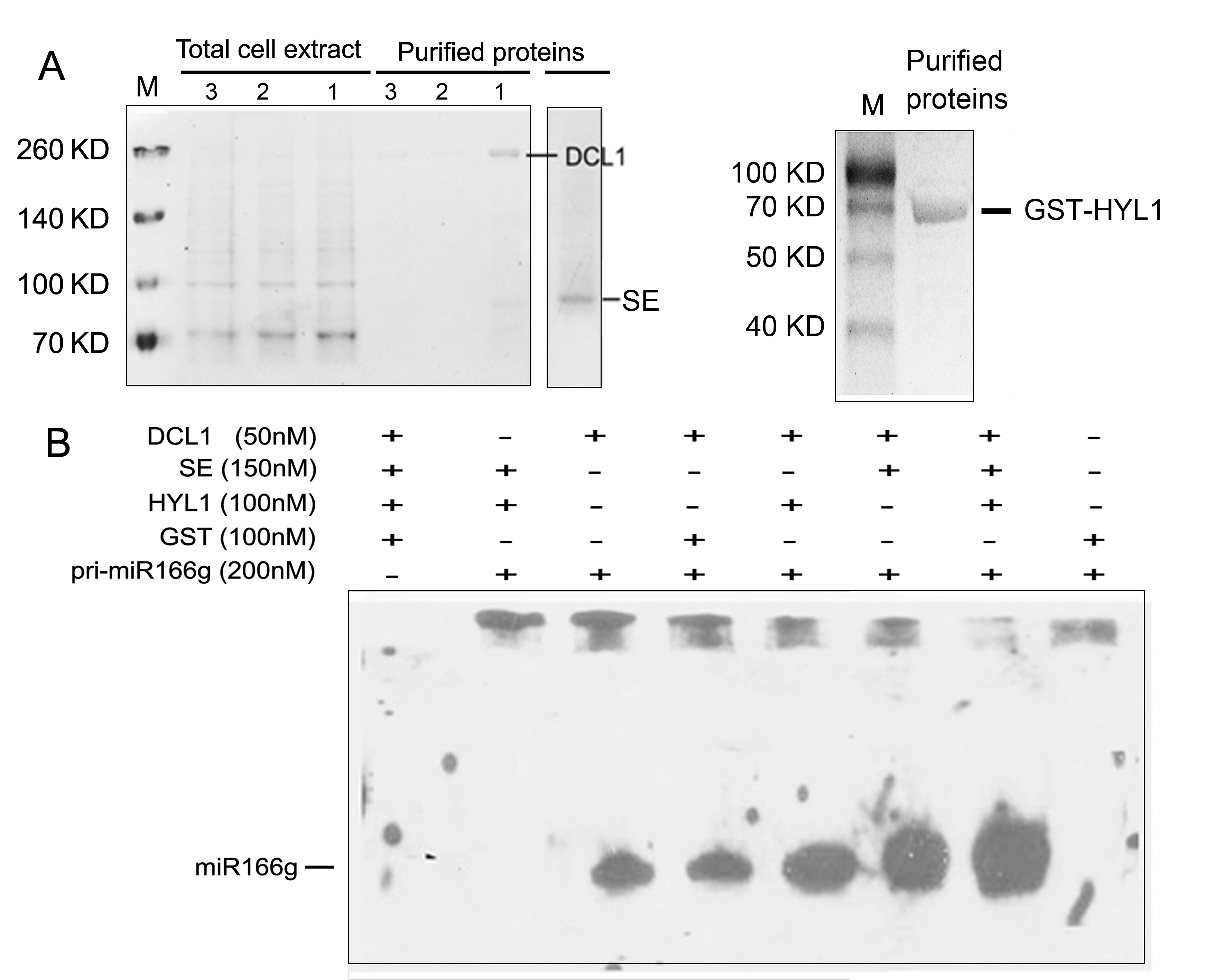


Figure S5. Purified DCL1 and SE proteins and *in vitro* miRNA processing system.

(A) Purified DCL1 and SE proteins isolated from HEK293 cells and HYL1 proteins from *Escherichia coli*. (B) Processing of pri-miR166g to mature miR166 in the *in vitro* miRNA processing system.


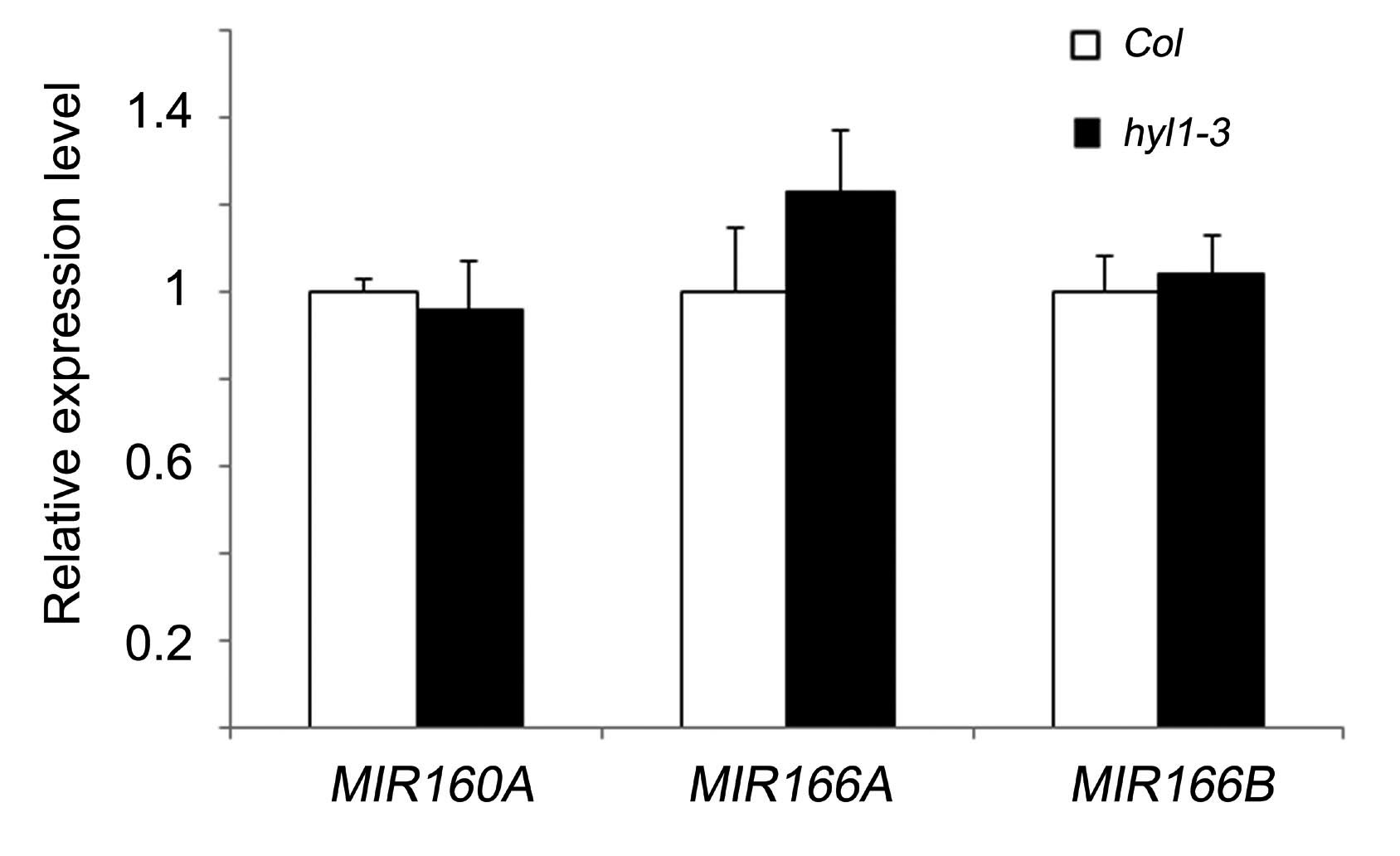
Figure S6. Primary transcript levels of three *MIRNA* genes showing their transcriptional activities in *hyl1-3* plants.

Table S1. Proteins identiﬁed to interact with HYL1 using yeast two-hybrid screening.

| Gene ID | Fragments interacted | Gene Functions |
| --- | --- | --- |
| AT1G09700 (HYL1) | 5’ UTR+ N-terminal | dsRNA-binding protein |
| AT1G28520 | 5’ UTR+ N-terminal | Zinc finger protein. |
| [AT2G27100](http://www.arabidopsis.org/servlets/TairObject?id=34452&type=locus) (SE) | C-terminal | Zinc finger protein. |
| AT2G21620 | 5’ UTR+ Full length CDS | Response to dessication |
| AT3G18035 | Middle Fragment | Linker histone like protein |
| AT5G14370 | 5’ UTR+ N-terminal | CCT motif family protein |

Table S2. Point mutations of the amino acids in dsRBD2 domain of HYL1.

| Amino Acids | Location | Substitutions | Expected changes |
| --- | --- | --- | --- |
| Cys(C) | 122 | Arg(R) | Dimerization of HYL1 |
| Arg(R) | 130 | Glu(E) | Dimerization of HYL1 |
| Thr(T) | 146 | Arg(R) | Dimerization of HYL1 |
| Thr(T) | 146 | Glu(E) | Dimerization of HYL1 |
| Gly(G) | 147 | Glu(E) | Dimerization of HYL1 |
| Ile(I) | 158 | Glu(E) | Control |
| Leu(L) | 165 | Glu(E) | Dimerization of HYL1 |
| Leu(L) | 166 | Glu(E) | Dimerization of HYL1 |
| Arg(R) | 151 | Glu(E) | Interaction with DCL1 or SE |
| Lys(K) | 154 | Glu(E) | Interaction with DCL1 or SE |
| Arg(R) | 162 | Glu(E) | Interaction with DCL1 or SE |

Table S3. Sequences of primers and probes used in this study.

| **Primers** | | **Sequences（5’-3’）** | | **Genes and RNAs** |
| --- | --- | --- | --- | --- |
| **Vector construction** | | | | |
| atHYL1-1S  atHYL1-252A | | ATGACCTCCAGTGAGCTAAGCCAATGTGTTT  TTTTGCTAATTCCCGGAGAG | | HYL1 |
| atHYL1-253S  atHYL1-513A | | CTCCAGTGAGCTAAGCCAATGTGTTT  GTCTGACTGGATCGCTAAAAGAGCA | | HYL1 |
| atHYL1-1S  atHYL1-1260A | | ATGACCTCCAGTGAGCTAAGCCAATGTGTTT  TTATGCGTGGCTTGCTTCTGT | | HYL1 |
| atHYL1-C122R-S  atHYL1-C122R-A | | CCATTGTATCAGAGACAGAAGGTCG  CGACCTTCTGTCTCTGATACAATGG | | HYL1 |
| atHYL1-T146R-S  atHYL1-T146R-A | | GGCATAAAGTACAGAGGAGCTGCAAC  GTTGCAGCTCCTCTGTACTTTATGCC | | HYL1 |
| atHYL1-R130E-S  atHYL1-R130E-A | | CTCTTGGGGAGGTTACACAATTCAC  GTGAATTGTGTAACCTCCCCAAGAG | | HYL1 |
| atHYL1-R162E-S  atHYL1-R162E-A | | GCGCTGGGGAGACTGCTCTTTTAGCG  CGCTAAAAGAGCAGTCTCCCCAGCGC | | HYL1 |
| atHYL1-R151E-S  atHYL1-R151E-A | | GCTGCAACAGAGACTAAAAAAGATGC  GCATCTTTTTTAGTCTCTGTTGCAGC | | HYL1 |
| atHYL1-K154E-S  atHYL1-K154E-A | | GAACTAAAGAGGATGCTGAGATTAGC  GCTAATCTCAGCATCCTCTTTAGTTC | | HYL1 |
| atHYL1-I158E-S  atHYL1-I158E-A | | GATGCTGAGGAGAGCGCTGGGAG  CTCCCAGCGCTCTCCTCAGCATC | | HYL1 |
| atHYL1-L165E-S  atHYL1-L165E-A | | GAACTGCTGAGTTAGCGATCCAGTC  CTCCCAGCGCTCTCCTCAGCATC | | HYL1 |
| atHYL1-L166E-S  atHYL1-L166E-A | | GAACTGCTCTTGAGGCGATCCAGTC  GACTGGATCGCCTCAAGAGCAGTTC | | HYL1 |
| atHYL1-G147E-S  atHYL1-G147E-A | | TCATAAAGTACACAGAGGCTGCAACA  ATAAAGTACACAGAGGCTGCAACAAGA | | HYL1 |
| atHYL1-T146E-S  atHYL1-T146E-A | | TAAAGTACGAGGGAGCTGCAACAAGA  ATAAAGTACGAGGGAGCTGCAACAAGA | | HYL1 |
| atSE-1S  atSE-2163A | | ATGGCCGATGTTAATCTTCCTC  CTACAAGCTCCTGTAATCAATAACG | | SE |
| atDCL1-1S  atDCL1-5733A | | ATGGTAATGGAGGATGAGCCTA  AGAAAAAGTTTTATTTAAAAGCTCAAG | | DCL1 |
| atpHYL1-S1  atpHYL1-A1 | | AAGAATTCAGCAACCATCTTCGTCGT  AAGGATTCTTTCACGAGCAGAATGT | | HYL1 |
| T7-pri-miR166g-1S  pri-miR166g-1A | | TAATACGACTCACTATAGGGCTATTAAACCAAAGTTACTAAATCGATG  AAACATATATAGGTTTTGAATGGGGTG | | pri-miR166g |
| T7-pri-miR167b1S  pri-miR167b-1A | | TAATACGACTCACTATAGGGATTTCTCCACTTCTTGAGCTTCC  CCGGTTTCGTGTAGTCAACTGTGTGCG | | pri-miR167b |
| **RT-PCR and real-time PCR** | | | | |
| ACTIN-S  ACTIN-A | | TGGCATCAYACTTTCTACAA  CCACCACTDAGCACAATGTT | | ACTIN |
| atTCP4-RT-S  atTCP4-RT-A | | AGGGTTTCTGTTCGCTCCTCCTAC  GTCGGTGGAGATGGATTGGTGAT | | TCP4 |
| atPHB-RT-S  atPHB-RT-A | | TTTCTATAGCAGAGGAGGCCC  AGGAGCATACATCTGCGTGT | | PHB |
| atREV-RT-S  atREV-RT-A | | TGCTCCACTTGTTCCCTC  TAGCCTTACGACCCGATT | | REV |
| atSPL9-RT-S  atSPL9-RT-A | | CAAGTGGAAGGTTGTGGGATG  GTCGCCAATTCCCTTGTAGCT | | SPL9 |
| atHYL1-RT-1S  atHYL1-RT-287A | | ATGACCTCCACTGATGTTTCCTC  ACAGGTTGTGAAACACATTGGC | | HYL1 |
| pri-miR156a-RT-S  pri-miR156a-RT-A | | CTCAAGTTCATTGCCATTTTTAGG  GAGAGATTGAGACATAGAGAACGAAGA | | pri-miR156a  pri-miR156a |
| pri-miR160a-RT-S  pri-miR160a-RT-A | | TGTATCTGTTCATGCATGGACCAG  GTGAGGTACCTCTAATCTCTTC | | pri-miR160 |
| pri-miR166a-RT-S  pri-miR166a-RT-A | | GGGGCTTTCTCTTTTGAGG  CGAAAGAGATCCAACATGAATAG | | pri-miR166a |
| pri-miR166b-RT-S  pri-miR166b-RT-A | | GATTTTTCTTTTGAGGGGACTGTTG  GATTTTTCTTTTGAGGGGACTGTTG | | pri-miR166b |
| pri-miR168a-RT-S  pri-miR168a-RT-A | | AGTAGAGTCTCACCATCGGGCT  TTACACCTCGAGGATCCGATT | | pri-miR168a |
| pri-miR172a-RT-S  pri-miR172a-RT-A | | CGATGCAGCATCATCAAGATTC  GAACTGAAATCTCGCGATCAGA | | pri-miR172a |
| pri-miR319a-RT-S  pri-miR319a-RT-A | | TCCAAACGCTCTATCTCTTCA  GCTTCCTTGAGTCCATTCACA | | pri-miR319a |
| MIR166A-S  MIR166A-T | | ccttcagatttcagatttgattaggg  agcaatgtagaaaagttca | | MIR166A  MIR166A |
| MIR160A-S  MIR160A-T | | accatgtatatgtcatgacgcatatac  aattgaaagaacggaatcagagagag | | MIR160a  MIR160a |
| MIR166B-S  MIR166B-T | | gggacgaacacatgagagatagataa  atatatcacatggattcatagat | | MIR166b  MIR166b |
| **5'-RACE PCR** | | | |  |
| pri-miR168a-339  pri-miR168a-321 | | AAATCAAAACCAAAGCAAAGATTC  GATTCAACATTTGGGCAAACAAAAGGAGAC | | pri-miR168a |
| pre-miR168a-90  pre-miR168a-74 | | GGTTTGTGAGCAGGGATTGGATCCC  TCACAAACCAATAAAGGTTTAAAAGTCACG | | pre-miR168a |
| pre-miR166g-(-5)  pre-miR166g-43 | | CCGAGAATCATTTAGAGTTTTGAGTTGGG  GTTAACGAATTACTCTCCATGA | | pre-miR166g |
| **Reverse transcription** | | | |  |
| MIR166A-R | tcatggtattgaatgatgatgacgatgatagtttt | | | MIR166a |
| MIR166B-R | gtcagagaaagagttaaagatcgaactaagtttta | | | MIR166b |
| MIR160A-R | catgtttgtcatatcatagcactttttacgagga | | | MIR160c |
| **Sequencing** | | | |  |
| pJR1-5S-1 | | GGAGAGGACAGGGTACC | |  |
| pCAMBIA-lacZ-A | | TGCTGCAAGGCGATTAAGTTGGGT | | pJR1 |
| T7-1S | | TAATACGACTCACTATAGGG | | T7 |
| atHYL1-dCAPs-S  atHYL1-dCAPs-A | | CCATTGTATCAGTGCCAGAAGGTCGAAACT  AGCATCTTTTTTAGTTCTTGTTGGAGCT | | G147E |
| **Probes** | | | |  |
| anti-miR166-biotin | | | GGGGAATGAAGCCTGGTCCGA | miR166 |
| anti-miR167-botin | | | TAGATCATGCTGGCAGCTTCA | miR167 |
| anti-miR168-biotin | | | TTCCCGACCTGCACCAAGCGA | miR168 |
| anti-miR319-biotin | | | AGGGAGCTCCCTTCAGTCCAA | miR319 |
| anti-miR156-biotin | | | GTGCTCACTCTCTTCTGTCA | miR156 |
| anti-miR172-biotin | | | ATGCAGCATCATCAAGATTCT | miR172 |
| anti-miR164-biotin | | | TGCACGTGCCCTGCTTCTCCA | miR164 |
| anti-U6-biotin | | | TCATCCTTGCGCAGGGGCCA | U6 |
